# Honeybees disrupt the structure and functionality of plant-pollinator networks

**DOI:** 10.1038/s41598-019-41271-5

**Published:** 2019-03-18

**Authors:** Alfredo Valido, María C. Rodríguez-Rodríguez, Pedro Jordano

**Affiliations:** 1 Department of Integrative Ecology, Estación Biológica de Doñana (EBD-CSIC), C/Americo Vespucio 26, La Cartuja, 41092 Sevilla Spain; 2 Present Address: Island Ecology and Evolution Research Group, Instituto de Productos Naturales y Agrobiología (IPNA-CSIC), C/ Astrofísico Francisco Sánchez 3, 38206 La Laguna, Tenerife Spain

## Abstract

The honeybee is the primary managed species worldwide for both crop pollination and honey production. Owing to beekeeping activity, its high relative abundance potentially affects the structure and functioning of pollination networks in natural ecosystems. Given that evidences about beekeeping impacts are restricted to observational studies of specific species and theoretical simulations, we still lack experimental data to test for their larger-scale impacts on biodiversity. Here we used a three-year field experiment in a natural ecosystem to compare the effects of pre- and post-establishment stages of beehives on the pollination network structure and plant reproductive success. Our results show that beekeeping reduces the diversity of wild pollinators and interaction links in the pollination networks. It disrupts their hierarchical structural organization causing the loss of interactions by generalist species, and also impairs pollination services by wild pollinators through reducing the reproductive success of those plant species highly visited by honeybees. High-density beekeeping in natural areas appears to have lasting, more serious negative impacts on biodiversity than was previously assumed.

## Introduction

The western honeybee (*Apis mellifera*) is an economically important species native to Eurasia and Africa, which has been introduced almost worldwide for crop pollination and honey production^1^. Except in Africa, most of their present-day populations are actually supported by the beekeeping activity^2^. The role of honeybees as pollinators is currently under debate^3–5^. On one hand, due to the global pollinator decline, honeybees are promoted to improve crop production^4,6^. Yet, on the other hand, they have been shown to supplement, rather than substitute, pollination services by wild insects^6,7^.

Beekeeping has globally increased by ∼45% during the last half century^8^, in such a way that *A*. *mellifera* is considered a “massively introduced managed species” in both its native and introduced range^9^. Some studies have shown that the expansion of this agroindustry affects mutualistic interactions, potentially disturbing the structure and functioning of pollination networks in natural ecosystems^9–15^. Alternatively, the addition of super-generalist species, such as *A*. *mellifera*, may increase the overall ‘cohesiveness’ of the mutualisitic networks because of its positive effects on network components such as nestedness, modularity, and redundancy of interactions^13,16,17^. However, given the difficulty in carrying out field experiments in absence of honeybees, these predictions remain untested, limiting our ability to predict if the structural networks change under beekeeping, and if so, the implications for plant reproductive success.

The honeybee is considered a super-generalist pollinator that monopolizes a sizeable fraction of floral resources^10,18^, and generally disrupts the interactions between wild pollinators and plants^10,19,20^. It promotes non-mutual dependences between partners^13^, and also increases both selfing and interspecific pollen deposition, impairing fruit- and seed-set^21,22^. Yet, the effects of honeybees on the overall plant-pollinator network structure and functioning remain largely unexplored in natural ecosystems (but see Magrach *et al*.^23^).

Here, we investigated the ecological influence of beekeeping by using a replicated, three-year (2007–2009) human-induced experiment in Teide National Park (Tenerife, Canary Islands). Up to 2,700 beehives are introduced there for honey exploitation at the peak of spring bloom. We compare the pre- and post-establishment stages of beehives on the pollination network structure, but also the consequences on plant reproductive success by using two complementary field experiments (comparing the reproductive outcome at individual level in five plant species under presence/absence of honeybees, and by using distance from apiaries as a proxy of the relative abundance of honeybees in one plant species; see below).

The setup of beehives allows us to collect an extensive and unique dataset to define a sequential contrast, with a pre-test/post-test comparison, characterized by the transition from a honeybee-free habitat (*pre*-period), to a situation where *A*. *mellifera* dominated the pollinator community (*apis*-period). However, in 2007, honeybees were not installed in the south-western sector of the National Park. Thus, these special conditions of absence of beehives allowed us the use of 2007 data as a *control*-year, with honeybees practically absent for the whole season (Methods, see also Supplementary Information).

Our hypotheses tests rely on contrasts of a battery of network parameters between the *pre*- and *apis*-periods (Methods, see also Supplementary Information for a description of the parameters used)^16,24,25^ which provide complementary non-redundant information to assess the effect of beekeeping on our systems. We thus predicted, under the beekeeping activity: *i*) a reduction in the complexity of the plant-pollinator web, quantifiable as a decrease in connectance (*C*), diversity of interactions (*H’*), and linkage density (*LD*), since some species and interactions are potentially lost through resource competition. These parameters relate to the density and diversity of intractions among species. *ii*) an increase in both nestedness (*N*) and weighted nestedness (*wtNODF*) because the honeybee, as a super-generalist, becomes a central node in the pollination networks, visiting both generalist and specialist plant species. Nestedness refers to a structural property of the interaction network whereby species with higher interaction specificity tend to interact with the supergeneralists, with interactions that form proper subsets of those recorded for more generalized species. *iii*) a decrease in the mutual dependence between interacting partners, in turn increasing the asymmetry of interactions (*ISA*) because the disproportionately high population density of honeybees due to the beekeeping activity would promote asymmetric interactions with native plant species; *iv*) a reduction in modularity (*M*), and number of modules (*nM*), but also in the topological role of wild species connecting among (*c coefficient*), and within (*z-score*) modules, since honeybees monopolize a substantial fraction of interactions and potentially dismantle the modular structure of the wild pollination assemblage. Modularity is a structural property of networks by which interactions tend to occur within subsets of species, with few of them occurring among subsets; *v*) network structural changes, reflected in an alteration of the eigenvalues spectra for the interaction matrices. Eigenvalue spectra allow assessing the differences in overall structure of interaction matrices by exploring the eigenvalue profiles, that vary according to how interactions are distributed among species (see Methods and Supplementary Information). And *vi*) a decline in pollination outcomes (fruit- and seed-set, and seed mass) because honeybees also tend to promote both selfing and interspecific pollen transfer, ultimately reducing seed set and seed quality (Methods, see also Supplementary Information).

## Results and Discussion

**Figure 1:**
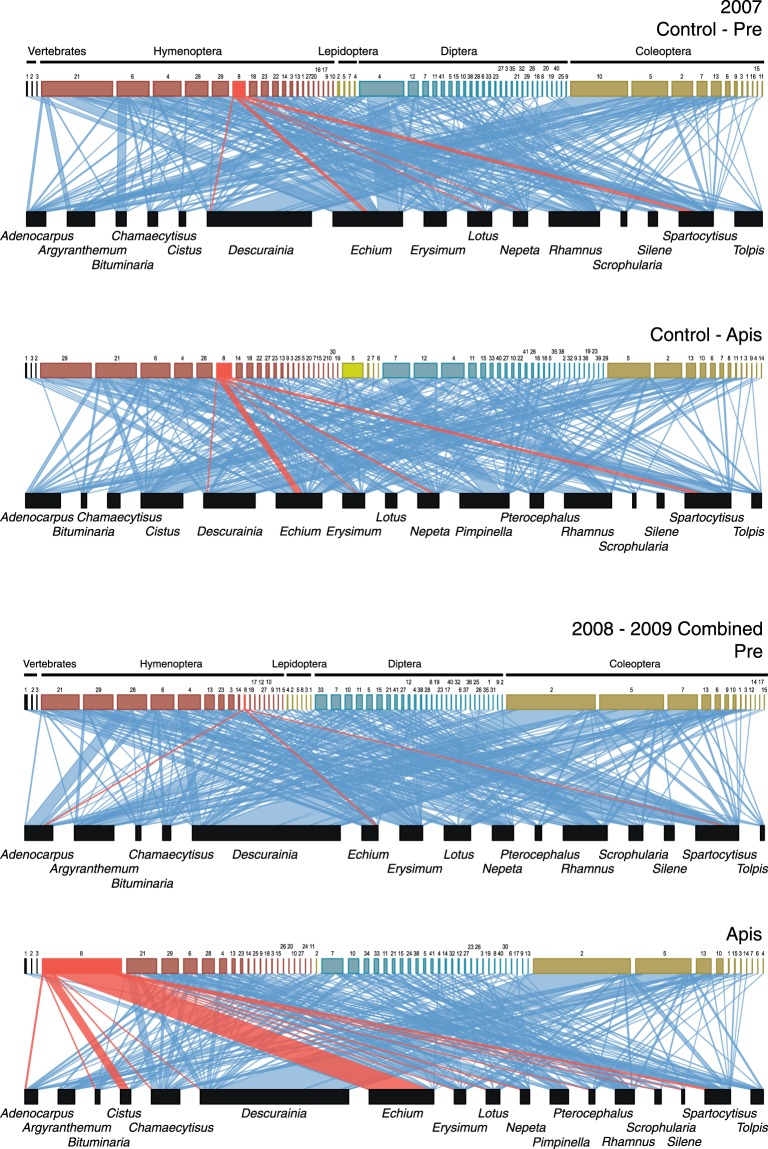
Pollination networks in Teide National Park in 2007 (*control*-year, with no beekeeping activities) and 2008–2009 (experimental years) combined. Size of boxes is proportional to the total number of visits recorded per species. Link width represents the frequency of observed plant-pollinator interactions. *A*. *mellifera* and its interactions are in red. See species identities and results separately per each year in Supplementary Information.

Across all three consecutive years, we recorded 23,096 mutualistic interactions corresponding to 545 distinctive links among 99 pollinator and 17 flowering plant species (Fig. 1; Supplementary Table S1). The sampling effort realised was sufficient to robustly characterise the number of pollinators for each experimental period and year (Supplementary Fig. S1). Honeybees visited 13 plant species, being also one of the most frequent flower visitors (9.2% of all visits), together with the beetles *Anaspis proteus* (15.8%) and *Attalus aenescens* (12.2%), and the bee *Hylaeus canariensis* (10.5%). Pooling all data, we obtained a connectance (*C*) of 32.4% (Supplementary Table S2). As reported in previous studies, honeybees become relatively well integrated into the existing pollination network^18,26^, by visiting a large number of plant species and with a high frequency of visits.

The onset of the beekeeping period triggered considerable shifts between the *pre-* and *apis-*periods, leading to a reduction in the number of pollinator species but also in interaction links. For example, we did not record 8 (in 2008), then 13 (2009) pollinator species through the *apis-* that were already observed in the *pre*-periods. Interestingly, 5 (2008; *Lasioglossum loetum*, *Bombus canariensis*, *Cyclirus webbianus*, *Nyctia lugubris*, *y Sphaeniscus filiolus*) and 9 (2009; *Gallotia galloti*, *Melecta curvispina*, *Dilophus beckeri*, *Sciaridae sp*., *Limnophora sp*., *Sphaeniscus filiolus*, *Bruchidius lichenicola*, *Attalus pellucidus*, and *Attalus monticola*) of these missing species were also detected through *control-apis* period (2007). Additionally, most plant species (9 in 2008 and 12 in 2009) were visited by a lower number of pollinator species through the *apis-*periods (Supplementary Tables S3A and S4). This trend was also seen in a significant decrease in both qualitative (12.9% and 14.5% in 2008 and 2009, respectively) and quantitative interactions links (18.1% and 9.91% in 2008 and 2009, respectively) interaction links per wild species (Supplementary Tables S3A and S4). Phenological differences between periods (the *pre-* period always precedes the *apis*-period) could explain these results, irrespective of beekeeping. However, when data from the *control*-year (2007) were analysed, we detected the opposite trend, an increase in both the number of pollinator species (5.8%), and the quantitative interaction links (14.6%) through the *control*-*apis*-period. Moreover, we obtained similar qualitative interaction link values (a difference of just 1.9%) in the comparison between *control*- periods (Supplementary Tables S3A and S4). This pattern was even more evident when comparing the number of vertebrates visiting *Echium wildpretii* (Boraginaceae) with the higher numbers during the *pre*- than *apis*-periods in both 2008 and 2009 (Supplementary Table S3B). Thus, as pointed out in several previous studies, the high relative abundance of honeybees owing to beekeeping suppressed flower visitation by wild pollinators due to exploitative competition^10,19–21,26–28^ as nectar standing crops are generally depleted by the massive presence of honeybees e.g.^20,21^. Yet our results reveal these negative consequences had greater far-reaching effects on the diversity of interactions (i.e. the whole pollination network structure), greater than expected from single-species consequences.

The reduction of both number of pollinator species and interaction links through *apis*-periods probably contributed substantially to the web simplification through shifts in network parameters. Consistent with our hypothesis, the pollinator web showed a significant lower connectance (*C*) under the beekeeping activity (P < 0.05). It is noticeable that the magnitude of *C* differences between experimental periods was also significantly higher in 2008–2009 than in the *control*-year (P < 0.001). However, beekeeping did not alter interaction diversity, linkage density or the interaction strength asymmetry (P > 0.05, Supplementary Fig. S3A and Table S2).

**Figure 2:**
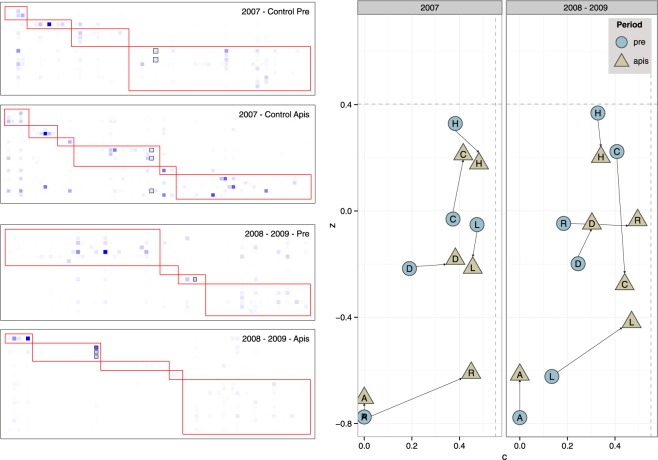
Modules (left) and species roles (right) for the control year (2007) and 2008–2009 combined. Species are sorted according to their assignment to modules, plants as rows and pollinators as columns. Darker squares indicate more frequent interactions. Squares with black outlines indicate honeybee. Trends in the topological role of pollinator species (right; pooled by Order) through *pre*- and *apis*-periods for the *control*-year (2007) and 2008–2009 combined. Roles are indicated by *c*, the fraction of interactions involving species in different modules, and *z*, the fraction of interactions with species in the same module. Dashed lines indicate the 0.95 percentile threshold values estimated on the species-specific data. H: Hymenoptera; C: Coleoptera; D: Diptera; R: Reptiles; L: Lepidotera; A: Aves. See species identities and results per pollinator species in Supplementary Information.

This network simplification prompted the question of how plant-pollinator interactions were then hierarchically organized after beekeeping. Our results showed a non-significant trend towards a more nested network (*N*); contrary to our expectations however, a significantly smaller weighted nestedness (*wtNODF*) (Supplementary Fig. S3A and Table S2), indicated that honeybees did not really overcompensate the lost pollinators and interaction links. Furthermore, the presence of honeybees significantly increased the modularity (*M* and *nM*) (Supplementary Table S2). The presence of honeybees seemed to dismantle the cohesively nested structure of the wild pollination assemblage, causing the loss of most interactions involving hub species (Fig. 2) and resulting in a higher modularity. For example, the native bees *Andrena chalcogastra*, *Colletes dimidiatus*, *Melecta curvispina*, and *Osmia canaria* reduced their interaction frequency by a 35.8% from *pre*- to *apis*-periods in 2008–2009, mostly losing among-module connector interactions (*c* coefficient; Supplementary Fig. S5A,B and Table S5). Given that these generalists contribute to the overall network connectivity, the loss of their interactions resulted in an increase in *M* and *nM*, also yielding the observed decrease in *wNODF*. Thus, our results show that beekeeping hits primarily those native supergeneralist species sharing floral resources (i.e. *Echium wildpretii*, *Spartocytisus supranubius*, *Nepeta teydea*, *Chamaecytisus proliferus*) with honeybees, resulting therefore in a loss of species that glue together the different modules of the network.

**Figure 3:**
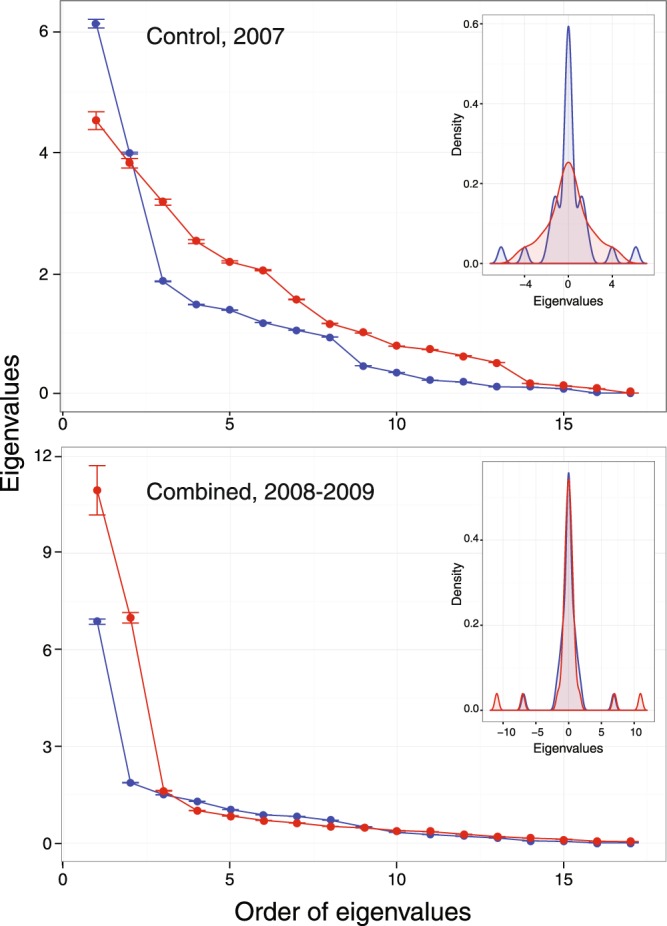
Rank ordered eigenvalues (with 95% confidence intervals) of the adjacency matrices for the *control*-year (2007, top) and experimental years (2008–2009 combined, bottom). The largest eigenvalue of the matrix is known as its spectral radius. Insets show the spectral graphs of the adjacency matrices for each period. Blue, *pre*-period; red, *apis*-period.

The changes detected in *wNODF*, *M*, and *nM* suggest structural modification of the overall pollination network (Fig. 3). According to this, we detected a higher leading eigenvalue (i.e. due to the dominance by *Apis*) (λ_1_ = 11.4) of the *apis*-period adjacency matrix compared to *pre*-period (λ_1_ = 7.0), for the pooled years 2008–2009. The situation was the reverse in the control year, 2007 (λ_1_ = 6.08 vs. λ_1_ = 4.45 in *control-pre-* and *control-apis* periods, respectively). In addition, the multiple “bumps” in the spectral graphs (Fig. 3 insets) are indicative of a distinctly disconnected pattern that reverses from the *pre*- to the *apis-*periods from 2007 to 2008–2009. This points to a consistent topological (i.e. distribution of links among nodes) and structural (i.e. how links are distributed internally) change in the overall pollination network, associated with honeybee overdominance: centralization of interactions by the honeybees, together with a loss of native supergeneralists causing a more modular assembly.

Taken together, our results regarding the specific and overall network descriptors reveal structural modifications of the plant-pollinator assemblages, driven by the beekeeping activitiy. Starting from a wild pollinator assemblage dominated by a distinct and diverse core of generalists, the beekeeping activity drives a comparative loss of wild pollinators, e.g. flower-visiting vertebrates practically disappear due to a nearly complete depletion of nectar by honeybees, and a selective reduction of the interactions by generalists that promote among-module cohesiveness. In fact, the presence of honeybees provides this re-centralization, yet with an impoverished diversity of pollinator taxa and interaction richness.

Many functional processes within ecosystems are directly related to the outcomes of species interactions^29–32^. The structural changes identified in the pollination networks were likely to have negative consequences on pollination functioning^33–35^. Indeed, those plant species highly visited by honeybees (*E*. *wildpretii* and *S*. *supranubius*) showed a significantly lower (P < 0.05) number of seeds/fruit through the *apis*-period, as expected (Table 1). Interestingly enough, fruit-set was also significantly higher through the *apis-*period in these two species (P < 0.001), possibly related to the higher (but not effective) relative visitation rate by honeybees. These results were also supported by the distance from apiaries gradient experiment carried out with *S*. *supranubius*. In this case, plants growing closer to apiaries produced a significantly lower number of seeds/fruit and heavier seeds than those at farther distances (P < 0.005, Supplementary Fig. S7). For example, the 41.8% and 30% of fruits sampled from plants nearest to apiaries (at 0 and 100 m distance classes, respectively) were empty (with only aborted seeds) compared to those collected at 500 m (3.1%) and 1000 (3.6%).

**Table 1:** Fruit-set and number seeds/fruit from selected plant species with contrasted incidence of honeybees.

Plant species (N° individual plants)	Relative visit rate by honeybees		Autogamy	Experimental time periods		*P value*
				*pre-*	*apis-*	
*Erysimum scoparium* (Brassicaceae) (N = 26)	Low	Fruit-set	45.71 ± 37.12 (322)	70.61 ± 16.26 (792)	67.14 ± 25 (333)	0.901
		N° seeds	1.83 ± 3.91 (154)	10.90 ± 7.98 (529)	14.09 ± 7.73 (229)	0.244
		A *vs*. nA		0.00 *vs*. 1.58	0.02 *vs*. 1.02	
*Scrophularia glabrata* (Scrophulariaceae) (N = 25)	Low	Fruit-set	6.38 ± 8.32 (430)	66.74 ± 17.28 (924)	64.10 ± 28.07 (645)	0.619
		N° seeds	55.70 ± 52.6 (31)	82.85 ± 45.45 (631)	93.61 ± 45.18 (380)	0.304
		A *vs*. nA		0.00 *vs*. 1.42	0.03 *vs*. 0.81	
*Adenocarpus viscosus* (Fabaceae) (N = 15)	Moderate	Fruit-set	0.74 ± 2.87 (110)	35.11 ± 16.13 (281)	38.21 ± 14.61 (312)	0.534
		N° seeds	0.07 ± 0.26 (1)	2.80 ± 1.15 (102)	2.43 ± 0.86 (123)	0.304
		A *vs*. nA		0.11 *vs*. 1.96	0.17 *vs*. 1.33	
*Echium wildpretii* (Boraginaceae) (N = 10)	High	Fruit-set	35.52 ± 5.54 (1627)	37.62 ± 11.77 (1582)	62.23 ± 19.46 (2316)	**0**.**001**
		N° seeds	1.77 ± 0.34 (553)	2.14 ± 0.49 (576)	1.68 ± 0.49 (1532)	**0**.**043**
		A *vs*. nA		0.00 *vs*. 0.80	8.04 *vs*. 0.41	
*Spartocytisus supranubius* (Fabaceae) (N = 25)	High	Fruit-set	0.00 ± 0.00 (820)	2.39 ± 3.38 (2182)	6.71 ± 7.43 (1441)	**0**.**006**
		N° seeds	—	2.19 ± 1.36 (54)	1.55 ± 1.3 (107)	**0**.**017**
		A *vs*. nA		0.00 *vs*. 2.85	1.13 *vs*. 0.94	

Comparative data were obtained from the same individual plant in two experimental periods (*pre*- and *apis*-). “A. *vs*. nA” indicates the averaged 5-min visit rates by *A*. *mellifera vs*. non-*Apis* pollinators. The number of individual plants, flowers and fruits sampled by species and experimental periods are shown in parentheses. Values are mean ± SD.

*P* values correspond to paired-*t* tests.

The high abundance of honeybees relative to non-*Apis* pollinators can explain the pattern along this distance gradient, given that the abundance of honeybees continuously decreases with increased distance from the beehives e.g.^36^. This is accompanied by a parallel increase in the diversity of wild bees^15^. The pollination effectiveness of honeybees relative to non-*Apis* pollinators varies widely across plant species^10,26^, possibly related to variation in selfing capacity, honeybee visitation rate, and also to the extensive reduction in wild pollinators visits because of beekeeping activity. However, it is well documented that a reduction in pollinator diversity alone can affect reproductive outcome in plants e.g.^29^. For example, Magrach *et al*.^23^ detected a decrease in seed-set in *Cistus crispus* (Cistaceae) in response to a high honeybee visitation rate, following honeybee spillover from a mass-flowering crop.

Increasing the presence of honeybees due to human beekeeping in natural areas (and also in nearest mass-flowering crop areas because of spillover of honeybees) can negatively affect the biodiversity of wild pollinators, ecosystem functioning, and ultimately their resistance to global environmental change^37–39^. By using a replicated comparative approach, our results offer evidence for the vulnerability of both the structure and functioning of the plant-pollinator networks by the beekeeping activity, since managed bees become relatively well integrated into the existing pollination networks. Beekeeping disrupted the generalist wild pollinators and their interaction links, generating significant changes in their hierarchical structural organization. Moreover, beekeeping impaired pollination services and plant reproductive success. However, given that this study was performed in only one ecosystem, and from an oceanic archipelago characterized by the human introduction of honeybees^40^, further research into both introduced and native range is necessary to assess the global implications of beekeeping. Our results suggest that the global beekeeping increase may have more serious and long-lasting negative impacts for natural ecosystems than is currently assumed.

## Methods

### Study site

The study was carried out through three consecutive flowering seasons (2007–2009) in a 6-ha plot within in the south-western sector of Teide National Park (Cementerio de los Tajinastes, 28°12′N, 16°38′W; 2,080 m a.s.l.; Tenerife, Canary Islands). This area consists of an high-altitudinal plateau area (189.9 km^2^) with Teide stratovolcano at is centre. It is characterized by an average annual temperature of 11.8 °C, 430 mm in precipitation (mainly between October-March), and around 13 days of snow per year^41^. The scrubland vegetation is dominated by *Spartocytisus supranubius* (Fabaceae), *Scrophularia glabrata* (Scrophulariaceae), *Erysimum scoparium*, *Descurainia bourgeauana* (Brassicaceae) and *Nepeta teydea* (Lamiaceae), among others^42^. The blooming period extends from early April to mid-June. Beekeeping activity is authorized within the National Park for honey production. Each spring, up to 2,700 beehives are installed in 18 apiaries (14 beehives/km^2^)^43–45^.

### Experimental Setup

Predicting and quantifying the impact of beekeeping remains controversial due to unavoidable limitations in the experimental design, e.g. the absence of a perfect control site without honeybees, and the difficulty in carrying out rigorous manipulative experiments in replicated field trials^10,46,47^. Additionally, the consequences of beekeeping are context-dependent, extending over vast areas and spatial scales, and are related to the distance from apiaries, number of beehives within the apiary, density of apiaries in the whole area, plant and pollinator community, and bloom densities, etc^9,15,23,26^. Thus, in order to establish two contrasting honeybee-abundance regimes (presence/absence of honeybees), we took some advantages of this National Park to achieve a field experiment with sufficient replication within these constraints.

First, beekeepers install the beehives, during 1–2 consecutive nights, in the middle of the flowering peak (early May). Given that no wild swarm of honeybees is currently present in this area, the day of installation sets a strong transition from a honeybee-free habitat during the first half of the flowering season (from April to early May; “*pre-*period”), to a situation where honeybees dominate the flower visitor community in the second half (from early May to mid-June; *apis*-period hereafter). The *pre-*periods were characterized by the practical absence of honeybees in the study plot, with no beehives installed within a radius of at least 4 km around. We had only sporadic records of honeybees, probably from beehives installed >4 km (Supplementary Table S3A). During the *apis-*periods, honeybees were relatively abundant in the study area due to the presence of ca. 400 beehives within a 4 km radius from the study plot.

Second, we experimentally set-up 10 beehives within our study plot in 2008 and 2009, mimicking the beekeeping set-up in this area. These additional beehives were installed at exactly the same date as the beekeepers did in the rest of the Park. Thus, the transition between the *pre*- and the *apis*- treatments reflects the actual setting of a native pollinator community invaded by an extremely high density of honeybees as a consequence of the beekeeping activity. In addition, a similar number of beehives are installed in the same 18 apiaries year by year (with permission from the Park authorities)^44,45^. Thus, by selecting the same study plot (and replicating over two years) we can avoid confounding factors such as distance from apiaries, number of beehives, and also the varying density of flowering plant species, which could affect the general conclusions.

Third, during 2007 the beekeepers did not install beehives in the south-western sector of the Park. During that spring, we obtained field data about pollination interactions practically without interference from honeybees (only 5% of total pollinator visits were recorded for *A*. *mellifera* in 2007; Supplementary Table S3A). Independently of honeybee presence, we might expect the studied community to differ somehow between the *pre*- and *apis*-periods just in terms of the actual seasonal dynamics (*apis*-periods were always after the *pre-*periods). Therefore, we used the 2007 data as a “*control*-treatment” for the seasonal ‘background’ changes between the *pre*- and *post*- situations (*control-pre-* and *control-apis* periods, hereafter), and contrasted 2007 data with those from the pooled (see Results in main text) and separately years (2008, 2009; Supplementary Information) to assess the consistency of trends in the replicated experimental scenario.

Despite the limited replication due to the characteristics and large scale of the beekeeping activities, we are confident that N = 2 with these previous specific advantages plus the extensive use of resampling schemes (see below) for hypothesis testing provided a realistic human-induced ecological experiment to assess the effects of beekeeping on: *i*) the structure of the plant-pollinator network due to changes in wild pollination interactions, and *ii*) their early consequences for plant reproductive output. Additionally, including 2007 as control was a way to account for random background noise as a feasible remedy for a discrepancy between predictions and results e.g.^48,49^.

### Sampling protocol

For each experimental period and year, we sampled floral visits during 8–13 consecutive days (Supplementary Table S3A) to construct both quantitative and qualitative matrices indicating the average visit rate and presence/absence of interactions of each pollinator (A) to each plant species (*P*). We recorded only the interactions where floral visitors contacted either the anthers or stigmas, thus acting as potential pollen vectors (“pollinators” hereafter). The interactions data were obtained from a minimum of 10 randomly-selected individuals per plant species from the whole flowering plant community (N = 17 plant species) (Supplementary Table S3A). Exceptions were rare plant species in the study area such as *Cistus symphytifolius* (N = 3 flowering individuals), *Rhamnus integrifolia* (N = 5), *Chamecytisus proliferus* (N = 6), and *Tolpis webbii* (N = 7). The selected individual plants bore enough open flowers and buds to ensure adequate pollinator sampling throughout the two periods, so that each plant received multiple observation censuses during both experimental periods. However, neighbouring individual plants were used whenever the focal plant did not have enough open flowers during the *apis*-periods. For example, for the 5 min censuses, the plant coincidence between periods was 55% (2007), 80% (2008) and 58% (2009) (Table S3A). Besides, this value was 100% for ‘spot-censuses’ (vertebrates) in *E*. *wildpretii* (Table S3B).

On each individual plant, we identified and counted all pollinators at a close distance (1–2 m) in 5-min censuses. The observations were carried out during the peak of pollinator activity (10:00–18:00 h), and climatic conditions were similar during periods within and across years. Each individual plant was sampled a minimum of 10 times per experimental period and year. Thus, we obtained an average of 86 ± 49 samples per plant species, period and year. By pooling all the three years we accumulated 8047 five-min censuses. Around 50% of censuses (N = 4367) were carried out during the *pre-*periods (Supplementary Table S3A). In order to record the full range of flower-visiting animal species, we complemented the focal-plant sampling with extra-census observations. To that end, we walked through the study plot and recorded those floral visits frequently under-detected in the systematic five-min censuses, mainly those by butterflies. These observations were included in the qualitative plant-pollinator interaction matrix. The sampling effort realised was sufficient to robustly characterise the number of pollinators according to plant species, for each experimental period and year (Supplementary Fig. S1).

Practically all the pollinators were identified in the field, whenever possible with the aid of a reference collection. Unknown insect species were captured and identified by specialist taxonomists (see Acknowledgements). Some vertebrate species frequently visit the flowers of *E*. *wildpretii*^18,20,43^. To include these mutualistic interactions, we used additional censuses with the observer located at >10 m (Supplementary Table S3B). Our aim was to avoid pursuing vertebrates when watching flowers from the close distances needed for the insect censuses. These vertebrate observations (“spot-censuses”) consisted of short (~1–2 min) visual inspections of *E*. *wildpretii* inflorescences (up to 2–3 m high) throughout the day with the help of binoculars. For this, we sampled the same 10 plants already used for five-min censuses. For the whole study, we completed 1,355 spot-censuses balanced across periods and years (Supplementary Table S3B), with around 50% of them (N = 671) done during *pre-*periods.

### Functional impact of honeybees

We designed two complementary field experiments to assess the functional consequences of beekeeping on plant reproductive success. First, within the study plot, we randomly selected 10–26 individual plants from each of five plant species with contrasting levels of high (*E*. *wildpretii* and *S*. *supranubius*), moderate (*A*. *viscosus*), and low (*E*. *scoparium* and *S*. *glabrata*) honeybee visitation (Table 1). On each individual plant we randomly selected 1–2 branches per period: *pre- vs*. *apis-*. The experimental branches used during the *pre-*period were bagged the day before the introduction of the beehives, and remained closed through the *apis*-period. During the *apis*-period we only used flowers that opened after beehive installation. To test for dependence on pollinators for successful fruit-set and number seeds/fruit we also selected a third set of branches per individual plant and covered them with mesh bags to exclude all pollinators. For each experimental branch, we counted all open flowers and collected all the resulting mature fruits to estimate fruit-set and number seeds/fruit per experimental period separately. In total, we counted 14,117 flowers and collected 5,002 ripe fruits from 101 individual plants (Table 1). This field experiment was carried out during the spring of 2009.

Second, during spring 2010, we set up a second field experiment to estimate the reproductive output of *S*. *supranubius*, depending on the distance from the beehives as a proxy of the relative abundance of *A*. *mellifera*^36^. For this, we selected five apiaries within the Park and marked linear transects from each, originating from the beehive location. Along each transect, we randomly selected individual plants roughly assigned to different distance categories from the nearest apiary (around 0, 100, 500, 1000, 2000, and 4000 m) and geo-referenced a total of 60, 45, 30, 34, 19, 31 individual plants, respectively. After blooming, we collected 50 ripe fruits per individual plant from five randomly selected branches. In total, 10,626 fruits from 219 individual plants were used to build two complementary datasets. The first dataset included all sampled fruits, from which the number of seeds per fruit (ovules effectively fertilized) were calculated. The second dataset corresponded to a sub-sample from the first dataset (2,838 fruits from 68 individual plants) but differing in the inclusion of the number of aborted ovules per fruit. This allowed us to control for mother plant effects along the distance gradient. From this dataset, the seed-set and the weight of individual mature seeds were calculated.

### Statistical analysis

We pooled all pollinator interactions to create six plant-pollinator networks, one per period and year. Each pollination network consisted of a weighted (quantitative) adjacency matrix indicating the average visit rate of each pollinator (A) to each plant species (*P*). Network sizes varied depending on the period and year (Supplementary Table S2). Before analysis, we assessed the completeness of our sampling effort by estimating interaction accumulation curves from the raw data of individual censuses^50^, using *vegan* R package^51^. Our goal was to contrast some pollination network parameters^16^, but also the overall structural descriptors such as the eigenvalue spectra^52,53^, between experimental periods. By using *bipartite R* package^24^, we estimated the next parameters:

#### Connectance (C)

This measures the fraction of interactions actually occurring, out of all the possible. Hence, *C* = *I*/(*PA)*, where *I* is the number of pairwise interactions present in the network, and *P* and *A* the number of plant and animal species, respectively. Since in the presence of honeybees some native interactions are lost by resource competition^19,20^, we would expect consistently lower *C* values during *apis*-periods. However, if honeybees already overcompensate the lost interactions, this value could be higher.

#### Shannon diversity of interactions (H’)

This parameter is related to the diversity of interactions or links (log of interactions) within the network relative to the total number of individuals. To calculate this, only interactions >0 are included. Since honeybees compete for floral resources with many pollinators and also disrupt wild pollinators and their interaction links with plants^19,20^, we would expect a significantly lower *H’* values during *apis*-periods.

#### Linkage density (LD)

This indicates the mean number of links per plant or pollinator species, but weighted by the average number of interactions across the species. Since honeybees contribute to disruption of pollination by native pollinators^19,20^, we would expect a reduction in the linkage density of the plant-pollinator web through *apis*-periods.

#### Nestedness (N)

This index indicates whether species with higher specificity of interactions actually interact with a subset of the species itself interacting with those showing more generalized interactions. The values of this matrix temperature-based parameter range between 0 (perfectly random) and 1 (perfect nestedness). Since honeybee, as super-generalist, becomes a central node in the pollination network, visiting both generalist and specialist plant species^54^, we would expected consistently higher *N* values through the *apis*-periods. An increase in *N* would confer the network a higher relative structural robustness against perturbations^16,55^. However, increasing nestedness would also increase interspecific pollen transfer, with potential negative implications for plant reproductive outcome, in turn reducing the ecological functionality of the system in terms of fruit- and seed-set, and individual seed mass (see below).

#### Weighted nestedness (wtNODF)

This metric is a version of the nestedness *NODF* index, but now incorporating information on the frequency at which plant-pollinator interactions occur^56^. We used the *wNODF* index to reduce the potential bias introduced when comparing different network sizes and shapes. A weighted-nested network is characterized by a proper ranking of interaction frequency, where mutualistic partners with more generalized interactions appear with higher frequency than those partners with higher specificity of interactions. The core of such an interaction matrix is characterized by a high frequency of interactions among the most generalized taxa. We expected honeybees to increase *wtNODF*, given their negative effect on low-specificity, rare interactions that were likely to disappear.

#### Interaction strength asymmetry (ISA)

This parameter quantifies the difference between the interaction strengths of partner species^13^. It corresponds to the average dependence of pollinators on plant species in relation to the dependence of plants on pollinator species. Positive values indicate greater dependence in the pollinator group and it is a measure of specialization across both trophic levels^24^. Through *apis*-periods, we would expect *ISA* to increase, because the disproportionately high population density of honeybees would promote asymmetric interactions with native plant species^13^.

#### Modularity (M) and number of modules (nM)

These parameters quantify the tendency of a network to be organized into distinct clusters, i.e. modular networks showing distinct subsets of taxa interacting more frequently among each other than with taxa in other modules^57^. We used the algorithm *QuanBiMo* (*bipartite* R package)^58^. Given that the estimation for the number of modules can vary between runs, the number of modules was calculated as the average (±SD) for 50 runs. We also checked for the consistency of module assignment for the different taxa and used the most frequent assignment for each species in the 50 runs. Since honeybees monopolize a substantial fraction of interactions, we would predict a decrease in both *M* and nM^17,25,59^.

#### Node position (c coefficient and z-score)

We compared the role of the different species (and functional groups) within the pollination networks by using two complementary parameters, *c* or participation coefficient (i.e. how many interactions occur with species in other modules) and *z* or within-module degree (i.e. how many interactions occur with species within their own module)^57^. We were interested in testing for significant displacements of the species’ positions on the *c*-*z* bivariate plane when contrasting the *pre*- and *apis*-periods. The consistency of these trends across both insect pollinator orders (and the vertebrate pollinators grouped together) and species level was explored by means of binomial tests on the sign of the trends for each species. Thus significant deviations from the binomial distribution would suggest a consistent decreasing or increasing trend in *c*-*z* values. We predicted a decrease in *c*-*z* values, especially for the hubs species, through *apis*-periods.

To compare the network parameters (*C*, *H’*, *LD*, *N*, *wNODF*, *ISA*, *M*, *nM*, z-score and *c* coefficient) between periods, two complementary analytical approaches were used: comparing periods within each year separately (2007 as control-year, 2008 and 2009; included in the Supplementary Information), and pooling all available data in the experimental years with honeybees (2008–2009, combined *pre*- and *apis*-periods; included in the maintext). For the comparisons with the pooled data, we used a subsample of 2007 data as our control treatment with the same sample size as the 2008 and 2009 *pre*-periods. We assessed if there was a significant change in network parameters between periods, using randomization tests. Each test involved randomizing of the experimental period (*pre*- vs. *apis*-period) in the adjacency matrices including data at individual plant level and recalculating the parameter values at each resample. The rationale is the following. First, we bootstrapped the raw, 5-min census, in the individual plant level dataset and built a randomized adjacency matrix for each *pre*-*apis* comparison at each resample. We kept the sample sizes originally obtained for each species. In each randomization run we generated two random networks by resampling the observed *pre*- and *apis*-period networks, to arrive at the parameter values for the two randomized networks. Then, we obtained the empirical, observed difference in parameter values between the *pre*- and the *apis*-period networks and compared the observed difference value with the frequency distribution of the differences found in the randomized resampling’s (N = 5000) of the adjacency matrices for the two periods. We obtained the *z*-score value of the observed difference relative to the distribution of randomized values and tested for its significance.

To compare the overall network structure between periods, we estimated the *eigenvalue spectra* of the observed adjacency matrices^52,53^. These are algebraic tools providing a thorough description better connected to the global network structure than different descriptive parameters^52^. The spectrum of a graph is the set of eigenvalues of the graph’s adjacency matrix. The largest eigenvalue, λ_1_, is also called the principal eigenvalue (spectral radius) of the graph. Along with the full eigenvalue spectrum, it can be used to detect differences interpretable in terms of overall structural changes^53^. Large eigenvalues indicate the presence of a core group of species, while a high frequency of zero eigenvalues indicates a high sharing of interactions among species in the network. To assess overall structural differences, we built ranked eigenvalue profiles, then estimated confidence intervals by resampling the raw adjacency matrix (as explained above) and calculating the eigenvalues for each iteration for the control year (2007) and the two additional experimental years (2008 and 2009), both separately (Supplementary Information) and for their combined data (in maintext). We expected honeybees to become a core species, generating low frequency of zero eigenvalues and lead to decreased web complexity (small spectral gap).

The results obtained from the first reproductive biology experiment (by using five representative plant species) were analysed with paired *t* tests. Within a focal individual plant, the fruit-set and number of seeds per fruit (log transformed) were compared between the two experimental periods. To test for among-treatment differences in reproductive success in *S*. *supranubius* according to beehive distances (second experiment), we fitted a generalized linear model (GLM) with Poisson (for number of seeds per fruit), binomial (for seed-set), and log (for individual seed mass) error distributions, using *multcomp* and *sandwich* R package^60,61^. To extract all pair-wise comparisons, we used a Tukey test. The number of ovules per fruit was included as a covariate (for the seed-set comparisons) in order to control for potential inter-individual variation, if any, in their reproductive capacity over the distance gradient. The number of seeds per fruit was also used as a covariate for individual seed mass comparisons. Data from individual flowers or fruits belonging to the same individual plant were averaged. Throughout the paper, all means are accompanied with their standard deviation unless otherwise indicated. All data analyses and related graphical representations were generated with R software version 3.1.1^62^.

## Supplementary information

Supplementary Information
