# Supplementary material for: Honeybees disrupt the structure and functionality of plant-pollinator networks
Source: Sci Rep. 2019 Mar 18;9:4711. doi: 10.1038/s41598-019-41271-5 (PMC6423295; doi:10.1038/s41598-019-41271-5)
Supplement: Supplementary file 1 — Supplementary Information [file 41598_2019_41271_MOESM1_ESM.pdf]

Supplementary Information for

Honeybees disrupt the structure and functionality of plant-pollinator networks

Alfredo Valido[1,*], María C. Rodríguez-Rodríguez and Pedro Jordano

Department of Integrative Ecology, Estación Biológica de Doñana (EBD-CSIC), C/ Americo Vespucio 26, La Cartuja,

41092 Sevilla, Spain

Telephone: +34 954466700

Fax: +34 954621125

[1] Present address: Island Ecology and Evolution Research Group, Instituto de Productos Naturales y Agrobiología (IPNA-CSIC), C/ Astrofísico Francisco Sánchez 3, 38206 La Laguna, Tenerife, Canary Islands, Spain.

* e-mail: avalido@ipna.csic.es

Supplementary Table S1 List of pollinator species recorded during three consecutive springs in the Teide National Park (Tenerife, Canary Islands). In total, 99 pollinator species were recorded (81 in 2007, 60 in 2008, and 75 in 2009). # indicate species codes (V: Vertebrata; H: Hymenoptera; D: Diptera; L: Lepidoptera; C: Coleoptera). Plants indicate total number of plant species visited each.

Order	Family	#	Species	2007	2008	2009	Plants
**Reptiles**	Lacertidae	V1	*Gallotia galloti*	1	1	1	3
**Aves**	Sylviidae	V2	*Phylloscopus canariensis*	1	1	1	1
**Aves**	Fringillidae	V3	*Serinus canarius*	1	1	1	1
**Hymenoptera**	Anthophoridae	H1	*Amegilla quadrifasciata*	1	-	-	4
**Hymenoptera**	Eumenidae	H2	*Ancistrocerus haematodes*	1	-	-	2
**Hymenoptera**	Andrenidae	H3	*Andrena chalcogastra*	1	1	1	11
**Hymenoptera**	Andrenidae	H4	*Andrena lineolata*	1	1	1	16
**Hymenoptera**	Megachilidae	H5	*Anthidium manicatum*	1	-	1	3
**Hymenoptera**	Anthophoridae	H6	*Anthophora alluaudi*	1	1	1	14
**Hymenoptera**	Braconidae	H7	*Apanteles* sp.	1	-	-	1
**Hymenoptera**	Apidae	H8	*Apis mellifera*	1	1	1	13
**Hymenoptera**	Apidae	H9	*Bombus canariensis*	1	1	1	4
**Hymenoptera**	Braconidae	H10	*Bracon* sp.	1	1	1	4
**Hymenoptera**	Braconidae	H11	*Braconidae* sp.	-	1	1	2
**Hymenoptera**	Formicidae	H12	*Camponotus feai*	-	-	1	1
**Hymenoptera**	Colletidae	H13	*Colletes dimidiatus*	1	1	1	11
**Hymenoptera**	Formicidae	H14	*Crematogaster alluaudi*	1	1	1	8
**Hymenoptera**	Ichneumonidae	H15	*Cryptus* sp.	1	1	1	4
**Hymenoptera**	Megachilidae	H16	*Dioxys atlantica*	1	-	-	1
**Hymenoptera**	Ichneumonidae	H17	*Dusona abdominator*	1	-	1	3
**Hymenoptera**	Anthophoridae	H18	*Eucera gracilipes*	1	1	1	12
**Hymenoptera**	Eupelmidae	H19	*Eupelmidae* sp.	1	-	-	1
**Hymenoptera**	Gasteruptiidae	H20	*Gasteruption canariae*	1	-	1	5
**Hymenoptera**	Colletidae	H21	*Hylaeus canariensis*	1	1	1	16
**Hymenoptera**	Halictidae	H22	*Lasioglossum arctifrons*	1	-	-	10
**Hymenoptera**	Halictidae	H23	*Lasioglossum loetum*	1	1	1	12
**Hymenoptera**	Formicidae	H24	*Lasius grandis*	-	-	1	1
**Hymenoptera**	Eumenidae	H25	*Leptochilus eatoni*	1	1	-	3
**Hymenoptera**	Megachilidae	H26	*Megachile canariensis*	-	-	1	2
**Hymenoptera**	Anthophoridae	H27	*Melecta curvispina*	1	1	1	6
**Hymenoptera**	Megachilidae	H28	*Osmia canaria*	1	1	1	15

Order	Family	#	Species	2007	2008	2009	#Plants
**Hymenoptera**	Formicidae	H29	*Plagiolepis barbara*	1	1	1	14
**Hymenoptera**	Ichneumonidae	H30	*Temelucha decorata*	1	-	-	1
**Lepidoptera**	Pieridae	L1	*Colias crocea*	-	1	-	1
**Lepidoptera**	Lycaenidae	L2	*Cyclyrius webbianus*	1	1	1	10
**Lepidoptera**	Pieridae	L3	*Euchloe belemia*	-	1	-	1
**Lepidoptera**	Geometridae	L4	*Eupithecia* sp.	1	1	1	5
**Lepidoptera**	Lycaenidae	L5	*Lycaena phlaeas*	1	-	1	7
**Lepidoptera**	Sphingidae	L6	*Macroglossum stellatarum*	1	-	-	1
**Lepidoptera**	Pieridae	L7	*Pieris rapae*	1	-	-	3
**Lepidoptera**	Nymphalidae	L8	*Vanessa cardui*	-	-	1	1
**Diptera**	Agromyzidae	D1	*Agromyzidae* sp.	-	-	1	1
**Diptera**	Bombyliidae	D2	*Anastoechus latifrons*	1	-	1	2
**Diptera**	Bombyliidae	D3	*Anthrax anthrax*	1	1	-	9
**Diptera**	Bibionidae	D4	*Bibio elmoi*	1	1	1	13
**Diptera**	Calliphoridae	D5	*Calliphora vicina*	1	1	1	10
**Diptera**	Tephritidae	D6	*Campiglossa reticulata*	1	1	1	3
**Diptera**	Mythicomyiidae	D7	*Cephalodromia* sp.	1	1	1	15
**Diptera**	Chloropidae	D8	*Chloropidae* sp.	1	1	1	5
**Diptera**	Syrphidae	D9	*Chrysotoxum triarcuatum*	1	1	1	3
**Diptera**	Mythicomyiidae	D10	*Cyrtosia canariensis*	1	1	1	8
**Diptera**	Anthomyiidae	D11	*Delia* spp. (2 species)	1	1	1	8
**Diptera**	Bibionidae	D12	*Dilophus beckeri*	1	1	1	14
**Diptera**	Empididae	D13	*Empis basilaris*	-	1	-	1
**Diptera**	Syrphidae	D14	*Eristalis tenax*	-	1	1	2
**Diptera**	Tachinidae	D15	*Estheria simonyi*	1	1	1	12
**Diptera**	Syrphidae	D16	*Eupeodes corollae*	1	-	-	5
**Diptera**	Bombyliidae	D17	*Exhyalanthrax simonae*	-	-	1	3
**Diptera**	Fanniidae	D18	*Fannia canicularis*	1	-	-	1
**Diptera**	Muscidae	D19	*Limnophora* sp.	1	1	1	5
**Diptera**	Agromyzidae	D20	*Liriomyza* sp.	1	-	-	1
**Diptera**	Calliphoridae	D21	*Lucilia sericata*	1	1	1	3
**Diptera**	Phoridae	D22	*Megaselia* sp.	1	-	-	1
**Diptera**	Sarcophagidae	D23	*Miltogramma aurifrons*	1	-	1	5
**Diptera**	Muscidae	D24	*Musca domestica*	-	-	1	1
**Diptera**	Conopidae	D25	*Myopa mixta*	1	1	-	2
**Diptera**	Sarcophagidae	D26	*Nyctia lugubris*	1	1	1	4
**Diptera**	Tachinidae	D27	*Peleteria ruficornis*	1	1	1	9

Order	Family	#	Species	2007	2008	2009	#Plants
**Diptera**	Muscidae	D28	*Phaonia sordidisquama*	1	1	1	4
**Diptera**	Conopidae	D29	*Physocephala biguttata*	1	-	-	2
**Diptera**	Tachinidae	D30	*Pseudogonia fasciata*	-	-	1	2
**Diptera**	Psychodidae	D31	*Psychodidae* sp.	-	-	1	1
**Diptera**	Tachinidae	D32	*Rondania insularis*	1	1	1	9
**Diptera**	Syrphidae	D33	*Scaeva albomaculata*	1	1	1	15
**Diptera**	Scatopsidae	D34	*Scatopsidae* sp.	-	-	1	2
**Diptera**	Sciaridae	D35	*Sciaridae* sp.	1	-	1	4
**Diptera**	Tephritidae	D36	*Sphaeniscus filiolus*	1	1	1	3
**Diptera**	Syrphidae	D37	*Sphaerophoria scripta*	-	-	1	1
**Diptera**	Tachinidae	D38	*Tachina canariensis*	1	1	1	8
**Diptera**	Tephritidae	D39	*Tephritidae* sp.	1	-	-	1
**Diptera**	Therevidae	D40	*Thereva teydea*	1	1	1	3
**Diptera**	Sarcophagidae	D41	*Wohlfahrtia bella*	1	1	1	8
**Coleoptera**	Buprestidae	C1	*Acmaeodera cisti*	1	1	1	7
**Coleoptera**	Scraptiidae	C2	*Anaspis proteus*	1	1	1	13
**Coleoptera**	Buprestidae	C3	*Anthaxia fernandezi*	1	1	1	5
**Coleoptera**	Dermestidae	C4	*Anthrenus minor*	1	-	1	2
**Coleoptera**	Malachiidae	C5	*Attalus aenescens*	1	1	1	17
**Coleoptera**	Malachiidae	C6	*Attalus monticola*	1	1	1	9
**Coleoptera**	Malachiidae	C7	*Attalus pellucidus*	1	1	1	13
**Coleoptera**	Anthicidae	C8	*Aulacoderus scydmaenoides*	1	-	-	1
**Coleoptera**	Bruchidae	C9	*Bruchidius lichenicola*	1	-	1	7
**Coleoptera**	Bruchidae	C10	*Bruchidius wollastoni*	1	1	1	6
**Coleoptera**	Curculionidae	C11	*Cionus griseus*	1	-	-	4
**Coleoptera**	Chrysomelidae	C12	*Cryptocephalus nitidicollis*	-	1	1	2
**Coleoptera**	Dasytidae	C13	*Dasytes israelsoni*	1	1	1	16
**Coleoptera**	Melyridae	C14	*Melyrosoma hirtum*	1	1	1	4
**Coleoptera**	Staphylinidae	C15	*Philorinum floricola*	1	1	1	3
**Coleoptera**	Staphylinidae	C16	*Staphylinidae* sp.	1	-	-	1
**Coleoptera**	Curculionidae	C17	*Tychius* sp.	-	-	1	1

Supplementary Table S2 Descriptive parameters of pollination networks in Teide National Park (Tenerife, Canary Islands) obtained throughout the three-years (2007-2009; and pooled all years), and for each experimental period per year in absence (*pre*-period) and massive presence (*apis*-period) of honeybees. In 2007, characterized by a practical absence of *A. mellifera*, we also set two temporal periods as control (*control-pre, control-apis*). For *Modularity* (M) and *number of modules* (*nM*) values are mean ± SD after 50 runs. See details in Material & Methods.

	**All years**	**2007**		**2008**		**2009**	
		control-pre	***control-apis***	***pre-***	***apis-***	***pre-***	***apis-***
Nº of plant species (p)	17	15	16	15	16	15	17
Nº of pollinator species (a)	99	65	69	52	44	64	51
Network size (M = a x p)	1683	975	1104	780	704	960	867
Nº of qualitative interactions	545	266	262	180	165	193	148
Connectance (C)	32.38	27.28	23.73	23.08	23.44	20.1	17.07
Nestedness (N)	29.44	19.79	19.86	19.39	23.41	21.39	20.23
Weighted nestedness (wNODF)	30.87	30.79	21.8	28.19	22.71	17.81	14.71
Interaction strength asymmetry (ISA)	0.10	0.72	0.71	0.74	0.71	0.69	0.69
Linkage density (LD)	6.86	5.93	5.04	4.82	4.01	4.27	3.18
Shannon diversity (H')	4.50	4.27	4.29	3.91	3.53	3.79	3.40
Modularity (M)	0.428 ± 0.039	0.325 ± 0.089	0.477 ± 0.047	0.373 ± 0.046	0.434 ± 0.026	0.314 ± 0.08	0.474 ± 0.089
Nº of modules (nM)	6.4 ± 0.9	4 ± 0.7	5.7 ± 0.6	4.6 ± 0.6	5.5 ± 0.8	4.1 ± 1	5.1 ± 0.8

Supplementary Table S3A Descriptive data of 5-min censuses under absence (*pre*-periods) and massive presence (*apis*-periods) of honeybees during three consecutive springs in Teide National Park (Tenerife, Canary Islands). In 2007, when *A. mellifera* was practically absent throughout the spring in our study plot, we set two temporal periods as control (*control-pre*, *control-apis*; see details in Material & Methods). The total number of interactions recorded (and percentage respect to the total for each year) is indicated per period.

	**2007**		**2008**		**2009**	
	control-pre	***control-apis***	***pre-***	***apis-***	***pre-***	***apis-***
Time span sampled	30 April-8May	9-16 May	1-10 May	12-21 May	2-14 May	18-28 May
Nº plant species (p)	15	16	15	16	15	17
Nº pollinator species (a)	65	69	52	44	64	51
Species richness (S= a + p)	80	85	67	60	79	68
Nº of individual plants censused	128	151	114	114	129	133
Coincidence of individual plants between periods	55%		80%		58%	
Total nº 5-min censuses realized	1405	1385	1372	1395	1590	900
Total nº quantitative interactions (5-min)	3678	4218	4127	4425	3654	2257
by Vertebrates (%)	6 (0.2)	1 (0.02)	17 (0.41)	28 (0.6)	10 (0.3)	1 (0.04)
by Apis mellifera (%)	184 (5)	209 (4.9)	16 (0.39)	910 (20.6)	10 (0.3)	759 (33.63)
by rest Hymenoptera (%)	1859 (50.5)	1789 (42.4)	1748 (42.36)	1092 (24.7)	1371(37.5)	317 (14.05)
by Diptera (%)	818 (22.2)	1224 (29)	309 (7.49)	294 (6.6)	653 (17.8)	409 (18.12)
by Coleoptera (%)	791 (21.5)	743 (17.6)	2033 (49.26)	2101 (47.5)	1599 (43.8)	770 (34.12)
by Lepidoptera (%)	20 (0.5)	252 (5.9)	4 (0.09)	0	11 (0.3)	1 (0.04)

Supplementary Table S3-B Descriptive data of vertebrate 'spot-censuses' done on 10 individual plants of *Echium wildpretii* (Boraginaceae) during three consecutive springs in Teide National Park (Tenerife, Canary Islands). Vertebrate pollinators included a lacertid lizard (*Gallotia galloti*), and two passerine birds (*Phylloscopus canariensis* and *Serinus canarius*). Data from 2008 and 2009 are separated into two temporal periods related to the absence (*pre*-periods) or massive presence (*apis*-periods) of honeybees. In 2007, when *A. mellifera* was practically absent throughout the spring in our study site, we differentiated two temporal periods as control (*control-pre*, *control-apis*; see details in Material & Methods).

	**2007**		**2008**		**2009**	
	control-pre	***control-apis***	***pre-***	***apis-***	***pre-***	***apis-***
Total nº spot-census realized	179	117	288	332	204	235
Total nº interactions with G. galloti	13	109	144	7	74	15
Total nº interactions with P. canariensis	7	5	25	3	26	0
Total nº interactions with S. canarius	0	0	24	1	4	3
Total nº interactions with birds	7	5	49	4	30	3
Total nº interactions with vertebrates	20	114	193	11	104	18
Vertebrate visit rate (mean ± SD)	0.13 ± 0.41	0.97 ± 1.34	0.67 ± 1.1	0.03 ± 0.2	0.51 ± 0.75	0.08 ± 0.28
Nº individual plants	10	10	10	10	10	10
Coincidence of individual plants between periods (%)	100		100		100	

Supplementary Table S4 Number of pollinator species registered on each plant species during three consecutive springs in Teide National Park (Tenerife, Canary Islands) under the absence (*pre*-period) and massive presence (*apis*-period) of honeybees (2008-2009 springs). For 2007, characterized by a practical absence of *A. mellifera* in our study plot, we distinguished two temporal periods as control (*control-pre*, *control-apis*; see details in Methods on Electronic Supplementary Material). The percentage of recorded species respect to the total for each year (81 in 2007; 60 in 2008; 75 in 2009; Table S1, Supporting Information) is indicated between brackets. '*#pollinators*' indicates the accumulated number of recorded species per plant species through the three studied years.

	**2007**		**2008**		**2009**		**#pollinators**
	control-pre	***control-apis***	***pre-***	***apis-***	***pre-***	***apis-***	
Adenocarpus viscosus (Fabaceae)	23 (30.8)	15 (22.1)	5 (8.3)	9 (15)	13 (20.3)	9 (18)	29
Argyranthemum tenerifae (Asteraceae)	26 (41.5)	-	22 (36.7)	11 (18.3)	18 (28.1)	10 (20)	41
Bituminaria bituminosa (Fabaceae)	5 (7.7)	6 (8.8)	10 (16.7)	9 (15)	7 (10.9)	4 (8)	19
Chamaecytisus proliferus (Fabaceae)	19 (15.4)	16 (23.5)	1 (1.7)	-	13 (28.1)	8 (6)	29
Cistus symphytifolius (Cistaceae)	22 (9.2)	22 (32.4)	-	14 (23.3)	-	7 (14)	29
Descurainia bourgeauana (Brassicaceae)	26 (29.2)	21 (30.9)	17 (28.3)	12 (20)	24 (37.5)	19 (38)	42
Echium wildpretii (Boraginaceae)	32 (33.9)	29 (42.7)	13 (21.7)	9 (15)	10 (20.3)	7 (14)	36
Erysimum scoparium (Brassicaceae)	31 (44.6)	24 (35.3)	17 (28.3)	17 (28.3)	16 (25)	11 (22)	40
Lotus campylocladus (Fabaceae)	19 (29.2)	5 (7.4)	12 (20)	8 (13.3)	10 (15.6)	7 (14)	29
Nepeta teydea (Lamiaceae)	26 (33.9)	20 (29.4)	15 (25)	10 (16.7)	15 (23.4)	9 (18)	38
Pimpinella cumbrae (Apiaceae)	-	21 (30.9)	-	3 (5)	-	6 (12)	27
Pterocephalus lasiospermus (Dipsacaceae)	-	24 (35.3)	5 (8.3)	8 (13.3)	7 (10.9)	11 (22)	38
Rhamnus integrifolia (Rhamnaceae)	22 (33.9)	7 (10.3)	19 (31.7)	13 (21.7)	13 (20.3)	15 (30)	39
Scrophularia glabrata (Scrophulariaceae)	14 (21.5)	10 (14.7)	6 (10)	3 (5)	10 (15.6)	4 (8)	23
Silene vulgaris (Caryophyllaceae)	4 (6.2)	1 (1.5)	6 (10)	7 (11.7)	4 (6.3)	2 (4)	12
Spartocytisus supranubius (Fabaceae)	37 (56.9)	30 (44.1)	27 (45)	22(36.7)	31 (48.4)	12 (24)	53
Tolpis webbii (Asteraceae)	10 (15.4)	12 (17.7)	5 (8.3)	10 (16.7)	2 (3.1)	7 (14)	21

Supplementary Table S5 Difference in the participation coefficient (Δc) and within-module degree (Δz) between *pre*- and *apis*-periods (*pre*- minus *apis*-) per pollinator species. Topological values were calculated from 2008-2009 combined data. Positive values indicate species with higher role connecting among (c) or within (z) modules through *pre*-periods. Pollinator species are group by order, except for Vertebrate. Within each order, species are sorted by decreasing Δc values. Species in boldface showed significantly lower c values under the presence of honeybees (*apis*-period) by using binomial test (see details in Material & Methods). See also figures 2 (maintext) and S5 (Supporting Information).

Order	#	Pollinator species	Δc (pre - apis)	Δz (pre-apis)
Vert-Aves	V2	*Phylloscopus canariensis*	0.0000000	-0.1937539
Vert-Aves	V3	*Serinus canarius*	0.0000000	-0.8270403
Vert-Rept	V1	*Gallotia galloti*	-0.3383838	0.0218467
**Hymenoptera**	**H3**	***Andrena chalcogastra***	**0.3503788**	**1.6732471**
**Hymenoptera**	**H27**	***Melecta curvispina***	**0.1994949**	**0.2669159**
**Hymenoptera**	**H11**	***Braconidae sp.***	**0.1969697**	**0.2472353**
**Hymenoptera**	**H9**	***Bombus canariensis***	**0.1111111**	**0.2096559**
**Hymenoptera**	**H28**	***Osmia canaria***	**0.1054924**	**-0.2639167**
**Hymenoptera**	**H13**	***Colletes dimidiatus***	**0.0740741**	**-0.2120282**
Hymenoptera	H1	*Amegilla quadrifasciata*	0.0000000	0.0000000
Hymenoptera	H2	*Ancistrocerus haematodes*	0.0000000	0.0000000
Hymenoptera	H5	*Anthidium manicatum*	0.0000000	-0.8270403
Hymenoptera	H7	*Apanteles sp.*	0.0000000	0.0000000
Hymenoptera	H12	*Camponotus feai*	0.0000000	-0.6502496
Hymenoptera	H16	*Dioxys atlantica*	0.0000000	0.0000000
Hymenoptera	H17	*Dusona abdominator*	0.0000000	0.0000000
Hymenoptera	H19	*Eupelmidae sp.*	0.0000000	0.0000000
Hymenoptera	H20	*Gasteruption canariae*	0.0000000	0.6543169
Hymenoptera	H22	*Lasioglossum arctifrons*	0.0000000	0.0000000
Hymenoptera	H24	*Lasius grandis*	0.0000000	0.6334324
Hymenoptera	H30	*Temelucha decorata*	0.0000000	0.0000000

Hymenoptera	H6	*Anthophora alluaudi*	-0.0150182	-0.0134454
Hymenoptera	H21	*Hylaeus canariensis*	-0.0396868	-0.1887345
Hymenoptera	H18	*Eucera gracilipes*	-0.0783839	0.1211488
Hymenoptera	H29	*Plagiolepis barbara*	-0.1000289	-0.1288708
Hymenoptera	H4	*Andrena lineolata*	-0.1198653	1.1256574
Hymenoptera	H8	*Apis mellifera*	-0.1733044	-2.6620538
Hymenoptera	H26	*Megachile canariensis*	-0.1818182	0.1788711
Hymenoptera	H23	*Lasioglossum loetum*	-0.2571892	0.9067189
Hymenoptera	H14	*Crematogaster alluaudi*	-0.2777778	0.1851121
Hymenoptera	H10	*Bracon sp.*	-0.3383838	-0.2617168
Hymenoptera	H15	*Cryptus sp.*	-0.4826038	-0.2492729
Hymenoptera	H25	*Leptochilus eatoni*	-0.5028058	0.4329302
**Lepidoptera**	**L4**	***Eupithecia sp.***	**0.3344557**	**-0.5239186**
Lepidoptera	L1	*Colias crocea*	0.0000000	-0.7524770
Lepidoptera	L3	*Euchloe belemia*	0.0000000	-0.8078621
Lepidoptera	L5	*Lycaena phlaeas*	0.0000000	-0.8078621
Lepidoptera	L6	*Macroglossum stellatarum*	0.0000000	0.0000000
Lepidoptera	L7	*Pieris rapae*	0.0000000	0.0000000
Lepidoptera	L8	*Vanessa cardui*	0.0000000	0.0000000
Lepidoptera	L2	*Cyclyrius webbianus*	-0.1414142	0.2425584
**Diptera**	**D8**	***Chloropidae sp***	**0.4654545**	**0.9328732**
**Diptera**	**D12**	***Dilophus beckeri***	**0.4511785**	**1.2349121**
**Diptera**	**D28**	***Phaonia sordidisquama***	**0.4419192**	**0.1192948**
**Diptera**	**D5**	***Calliphora vicina***	**0.1400742**	**1.3357965**
**Diptera**	**D19**	***Limnophora sp.***	**0.1374859**	**-0.0003736**
**Diptera**	**D15**	***Estheria simonyi***	**0.1146885**	**0.4950113**
**Diptera**	**D7**	***Cephalodromia sp.***	**0.0829649**	**-1.6934254**
**Diptera**	**D41**	***Wohlfahrtia bella***	**0.0050505**	**-0.6032783**
Diptera	D1	*Agromyzidae sp.*	0.0000000	0.0000000
Diptera	D2	*Anastoechus latifrons*	0.0000000	0.0000000
Diptera	D9	*Chrysotoxum triarcuatum*	0.0000000	-0.0277687
Diptera	D13	*Empis basilaris*	0.0000000	0.6332864
Diptera	D16	*Eupeodes corollae*	0.0000000	0.0000000
Diptera	D18	*Fannia canicularis*	0.0000000	0.0000000
Diptera	D20	*Liriomyza sp.*	0.0000000	0.0000000
Diptera	D22	*Megaselia sp.*	0.0000000	0.0000000
Diptera	D24	*Musca domestica*	0.0000000	0.7223893
Diptera	D25	*Myopa mixta*	0.0000000	-0.7973289

Diptera	D29	*Physocephala biguttata*	0.0000000	0.0000000
Diptera	D31	*Psychodidae sp.*	0.0000000	0.0000000
Diptera	D35	*Sciaridae sp.*	0.0000000	-0.9199442
Diptera	D36	*Sphaeniscus filiolus*	0.0000000	-0.7224066
Diptera	D37	*Sphaerophoria scripta*	0.0000000	-0.9240375
Diptera	D39	*Tephritidae sp.*	0.0000000	0.0000000
Diptera	D33	*Scaeva albomaculata*	-0.0098773	1.6516677
Diptera	D10	*Cyrtosia canariensis*	-0.0661637	-1.0385188
Diptera	D6	*Campiglossa reticulata*	-0.0858586	-0.2686548
Diptera	D17	*Exhyalanthrax simonae*	-0.0909091	-0.4275282
Diptera	D34	*Scatopsidae sp.*	-0.1060606	0.3585458
Diptera	D3	*Anthrax anthrax*	-0.1167228	-0.0072875
Diptera	D11	*Delia spp (2 species)*	-0.1201796	-1.0012273
Diptera	D21	*Lucilia sericata*	-0.1313131	-0.0841964
Diptera	D40	*Thereva teydea*	-0.1795735	-0.8145835
Diptera	D4	*Bibio elmoi*	-0.1993547	-1.1186693
Diptera	D27	*Peleteria ruficornis*	-0.2111111	0.5868842
Diptera	D23	*Miltogramma aurifrons*	-0.2878788	-0.5002055
Diptera	D30	*Pseudogonia fasciata*	-0.2878788	0.3071132
Diptera	D26	*Nyctia lugubris*	-0.3131313	-0.1763092
Diptera	D38	*Tachina canariensis*	-0.3243547	-0.3372174
Diptera	D14	*Eristalis tenax*	-0.4848485	0.6164558
Diptera	D32	*Rondania insularis*	-0.6313131	-0.5300534
**Coleoptera**	**C12**	***Cryptocephalus nitidicollis***	**0.4747475**	**-0.5089971**
**Coleoptera**	**C6**	***Attalus monticola***	**0.4468687**	**1.6764419**
**Coleoptera**	**C9**	***Bruchidius lichenicola***	**0.3344557**	**-0.2907843**
Coleoptera	C4	*Anthrenus minor*	0.0000000	0.6543169
Coleoptera	C8	*Aulacoderus scydmaenoides*	0.0000000	0.0000000
Coleoptera	C11	*Cionus griseus*	0.0000000	0.0000000
Coleoptera	C16	*Staphylinidae sp.*	0.0000000	0.0000000
Coleoptera	C17	*Tychius sp.*	0.0000000	0.0000000
Coleoptera	C2	*Anaspis proteus*	-0.0029306	1.3114811
Coleoptera	C3	*Anthaxia fernandezi*	-0.0112234	0.0658528
Coleoptera	C7	*Attalus pellucidus*	-0.0233972	1.7058109
Coleoptera	C13	*Dasytes israelsoni*	-0.0275761	-0.4417457
Coleoptera	C5	*Attalus aenescens*	-0.0447531	1.2445736
Coleoptera	C1	*Acmaeodera cisti*	-0.0576599	0.0020713
Coleoptera	C10	*Bruchidius wollastoni*	-0.1691919	0.4180418

Coleoptera	C15	*Philorinum floricola*	-0.4040404	-0.2198068
Coleoptera	C14	*Melyrosoma hirtum*	-0.4646465	-0.4114923

Supplementary Figure S1 Representation of cumulative number of pollinator species recorded for each experimental period (*pre*- and *apis*-periods) and year in Teide National Park (Tenerife, Canary Islands) in 2007 (*control*-year) and 2008 and 2009 (*experimental*-years), in relation to increasing sampling effort. According to this, the sampling effort realised was sufficient to robustly characterise the number of pollinators for each experimental period and year (see also tables S1, S2 and S3 for the species list and number of pollination interactions recorded for each period and year).

Supplementary Figure S2 Pollination networks in Teide National Park (Tenerife, Canary Islands) obtained in the *control*- (2007; A) and *experimental*-years (2008; B) (2009; C). The box size corresponds to the total number of flower visits recorded per pollinator species. Link width represents the frequency of observed interactions. Honeybees, and its interactions, are indicated in red. See species identities in Table S1.

Supplementary Figure S3 Observed values for each network parameter (*C*, *N*, *wNODF*, *ISA*, *H'*, *LD*) regarding the difference between the *pre*- and *apis*-period values (red line), compared with the frequency distribution of the differences found in the randomized resamplings (N= 5000) of the adjacency matrices for the two combined *experimental*-years (2008-2009; A), and for each year separately (2007; B), (2008; C), (2009; D). The significance values for the *z*-score transformed values of the differences are also included.

Supplementary Figure S4 Detected modules for each experimental period (*pre*- and *apis*-periods) and years (2007; A), (2008; B), (2009; C) and *experimental*-years combined (2008-

2009; D). Species are sorted according to their modular position, plants as rows and pollinators as columns. Darker squares indicate more frequent interactions.

Supplementary Figure S5 Changes in the topological role of pollinator species, pooled by Order (**A**) and species (**B**) for the *control*-year (2007) and experimental-years separately (2008, 2009) and combined (2008-2009), connecting across (*c coefficient*) and within (*z-score*) modules from *pre*- to *apis*-periods. H: Hymenoptera; C: Coleoptera; D: Diptera; R: Reptiles; L: Lepidotera; A: Aves. See species identities in Table S1 and results per pollinator species in Supplementary. The significant displacements of hub bee species (*A. chalcogastra*, *C. dimidiatus*, *M. curvispina,* and *O. canaria*) positions on the *c-z* bivariate plane are also indicated. See also Table S5.

Supplementary Figure S6 Rank ordered eigenvalues of the adjacency matrices for the 2008 and 2009 seasons separately (left). The largest eigenvalue of the matrix is known as its spectral radius; we obtained the 95% confidence intervals (bars) for eigenvalues by resampling the raw interaction matrix of individual plant data. Blue, *pre*-period; red, *apis*-period. Right panels show the spectral graphs of the adjacency matrices for the *pre*- (blue) and *apis*- (red) periods. The spectra are symmetric about the origin because the adjacency matrix of a binary bipartite graph with |S| nodes has |S| eigenvalues[63].

Supplementary Figure S7 (**A**) Variation in the number of seeds per fruit of *Spartocytisus supranubius* (Fabaceae) (N= 10,626 fruits from 219 individual plants) along a distance to nearest apiary gradient in Teide National Park (Tenerife, Canary Islands). The numbers of individual plants used per distance class are: 60 (0 m), 45 (100 m), 30 (500 m), 34 (1000 m), 19 (2000), and 31 (4000 m). Significant statistical differences among distances are represented by different subscript letters (GLM with Poisson error family). (**B**) Individual

seed mass of *S. supranubius* (N= 2838 fruits from 68 individual plants) along a distance to nearest apiary gradient. The number of individual plants used per distance class are: 15 (0 m), 10 (100 m), 10 (500 m), 9 (1000 m), 14 (2000), and 10 (4000 m). Significant statistical differences among distances are represented by different subscript letters (GLM with log transformation and using number of seeds per fruit as covariate). Data from fruits belonging to the same individual plant were averaged.

Supplementary Figure S8 Distribution of apiaries and the studied plot, within Teide National Park (Tenerife)[44-45]. Map data: Google Earth, Grafcan (2018).

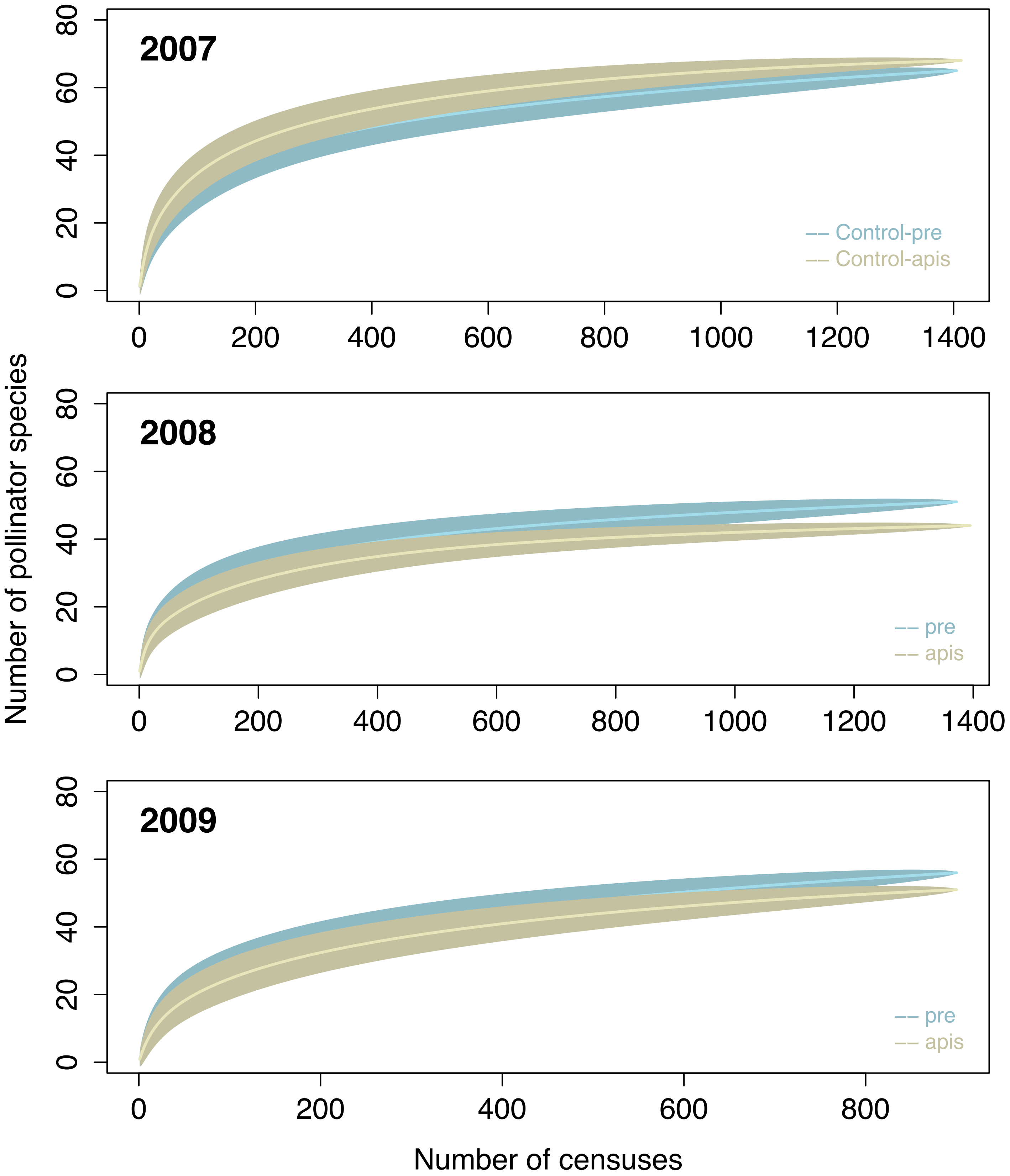
2007
Control-pre
Control-apis
2008
pre
apis
2009
pre
apis
Number of pollinator species
Number of censuses
0
20
40
60
80
0
200
400
600
800
1000
1200
1400

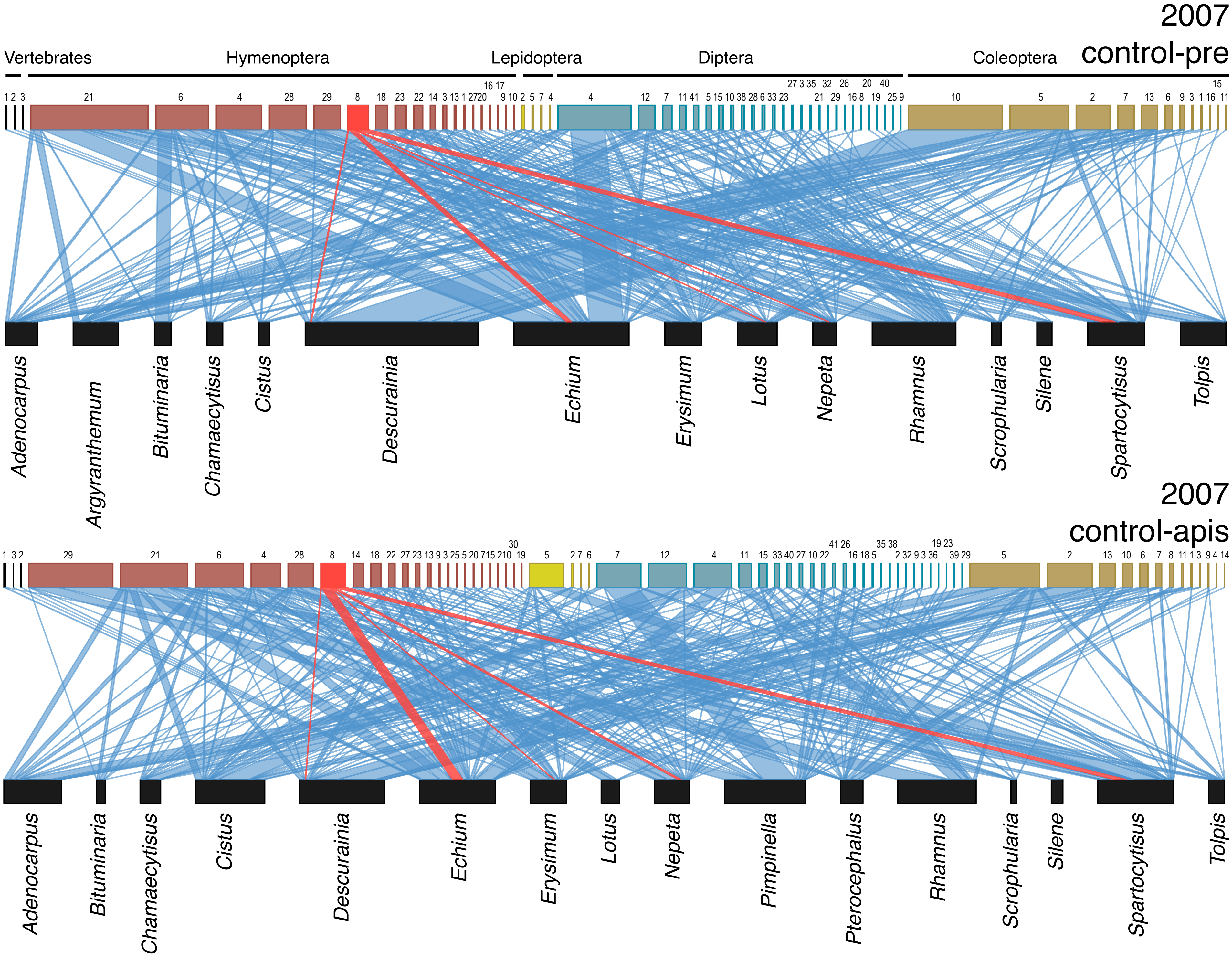
2007
control-pre
Vertebrates
Hymenoptera
Lepidoptera
Diptera
Coleoptera
Adenocarpus
Argyranthemum
Bituminaria
Chamaecytisus
Cistus
Descurainia
Echium
Erysimum
Lotus
Nepeta
Rhamnus
Scrophularia
Silene
Spartocytisus
Tolpis
2007
control-apis
Adenocarpus
Bituminaria
Chamaecytisus
Cistus
Descurainia
Echium
Erysimum
Lotus
Nepeta
Pimpinella
Pterocephalus
Rhamnus
Scrophularia
Silene
Spartocytisus
Tolpis

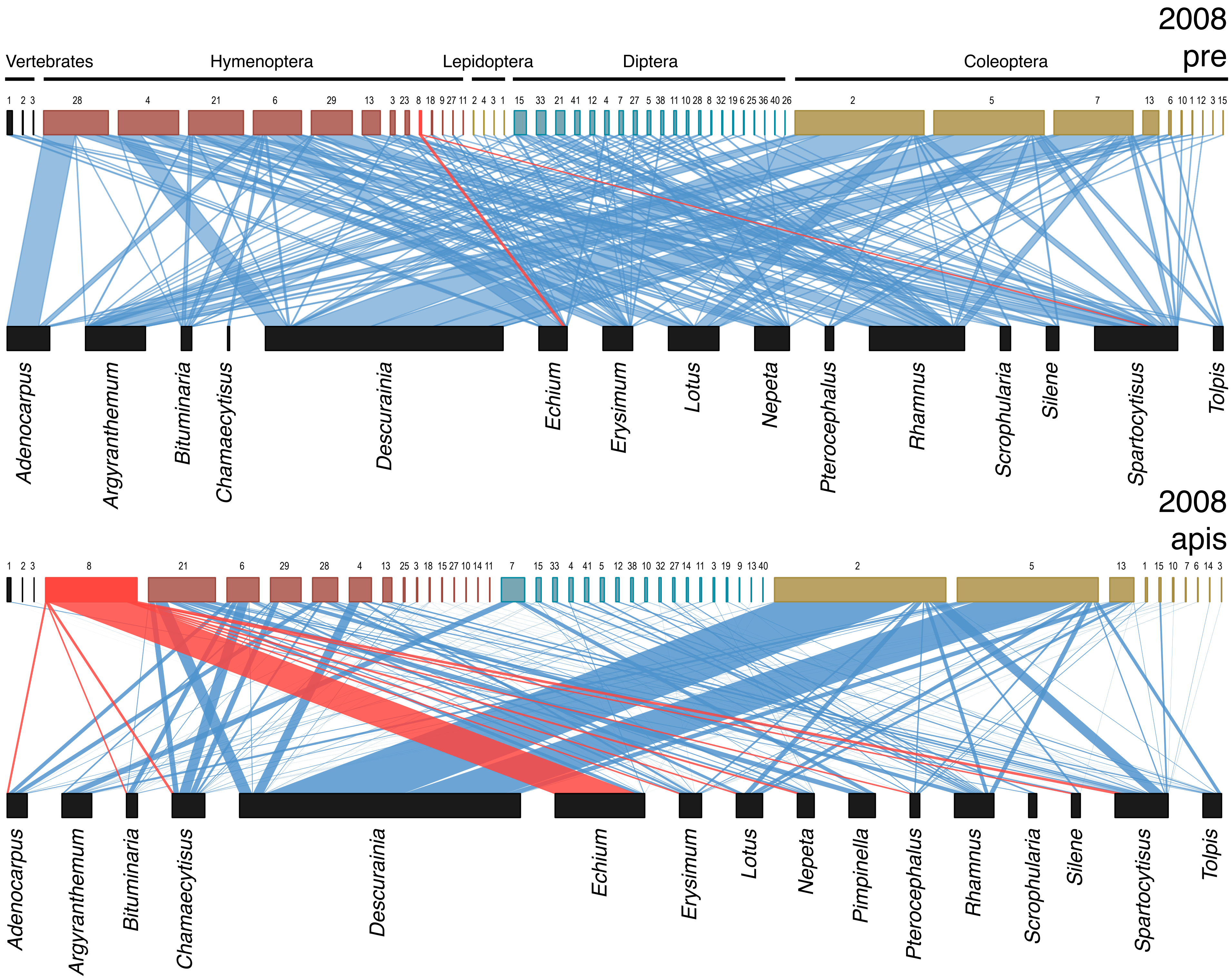
2008
pre
Vertebrates
Hymenoptera
Lepidoptera
Diptera
Coleoptera
1 2 3 28 4 21 6 29 13 3 23 8 18 9 27 11 2 4 3 1 15 33 21 41 12 4 7 27 5 38 11 10 28 8 32 19 6 25 36 40 26 2 5 7 13 6 10 1 12 3 15
Adenocarpus
Argyranthemum
Bituminaria
Chamaecytisus
Descurainia
Echium
Erysimum
Lotus
Nepeta
Pterocephalus
Rhamnus
Scrophularia
Silene
Spartocytisus
Tolpis
2008
apis
1 2 3 8 21 6 29 28 4 13 25 3 18 15 27 10 14 11 7 15 33 4 41 5 12 38 10 32 27 14 11 3 19 9 13 40 2 5 13 1 15 10 7 6 14 3
Adenocarpus
Argyranthemum
Bituminaria
Chamaecytisus
Descurainia
Echium
Erysimum
Lotus
Nepeta
Pimpinella
Pterocephalus
Rhamnus
Scrophularia
Silene
Spartocytisus
Tolpis

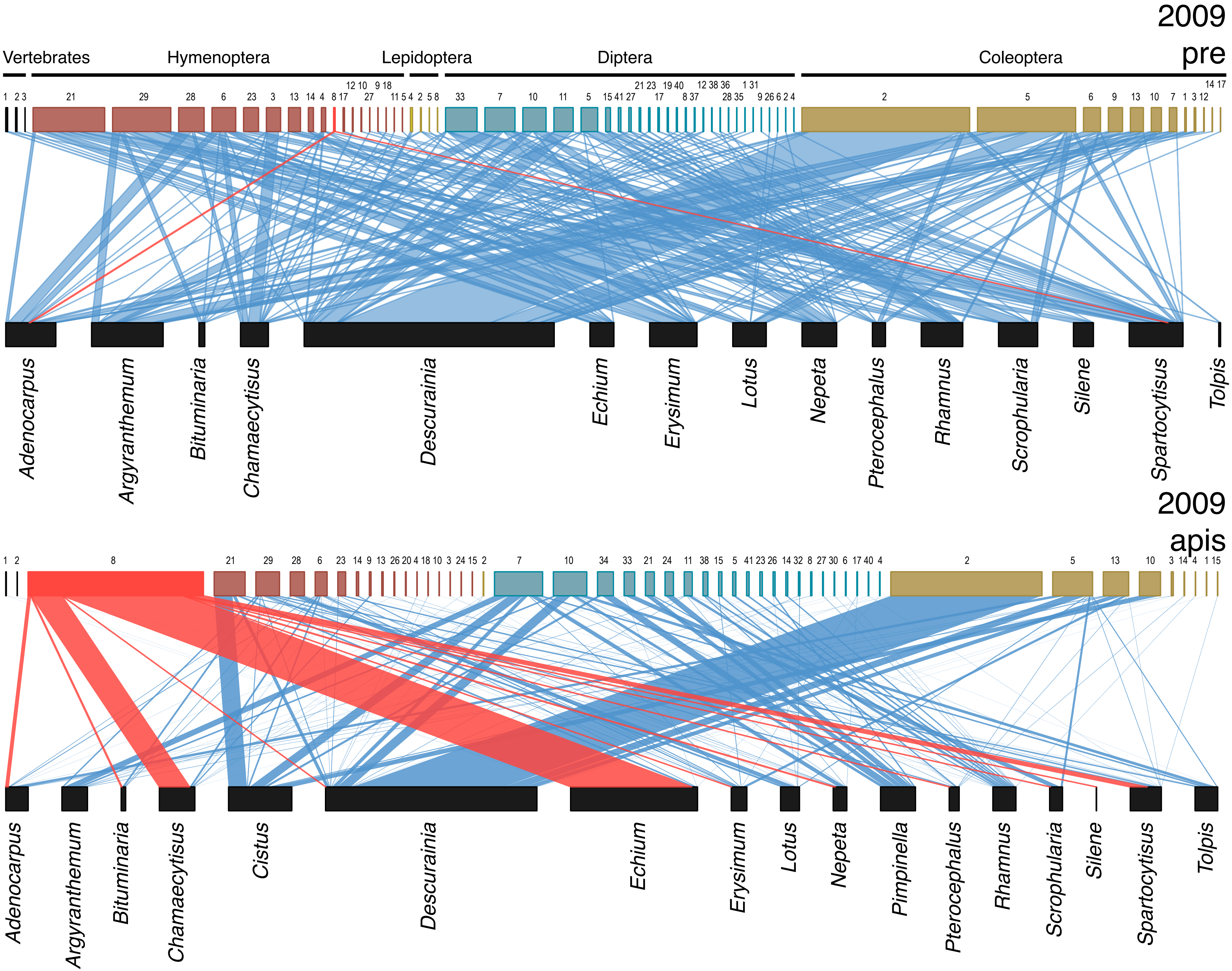
2009
pre
Vertebrates
Hymenoptera
Lepidoptera
Diptera
Coleoptera
Adenocarpus
Argyranthemum
Bituminaria
Chamaecytisus
Descurainia
Echium
Erysimum
Lotus
Nepeta
Pterocephalus
Rhamnus
Scrophularia
Silene
Spartocytisus
Tolpis
2009
apis
Adenocarpus
Argyranthemum
Bituminaria
Chamaecytisus
Cistus
Descurainia
Echium
Erysimum
Lotus
Nepeta
Pimpinella
Pterocephalus
Rhamnus
Scrophularia
Silene
Spartocytisus
Tolpis

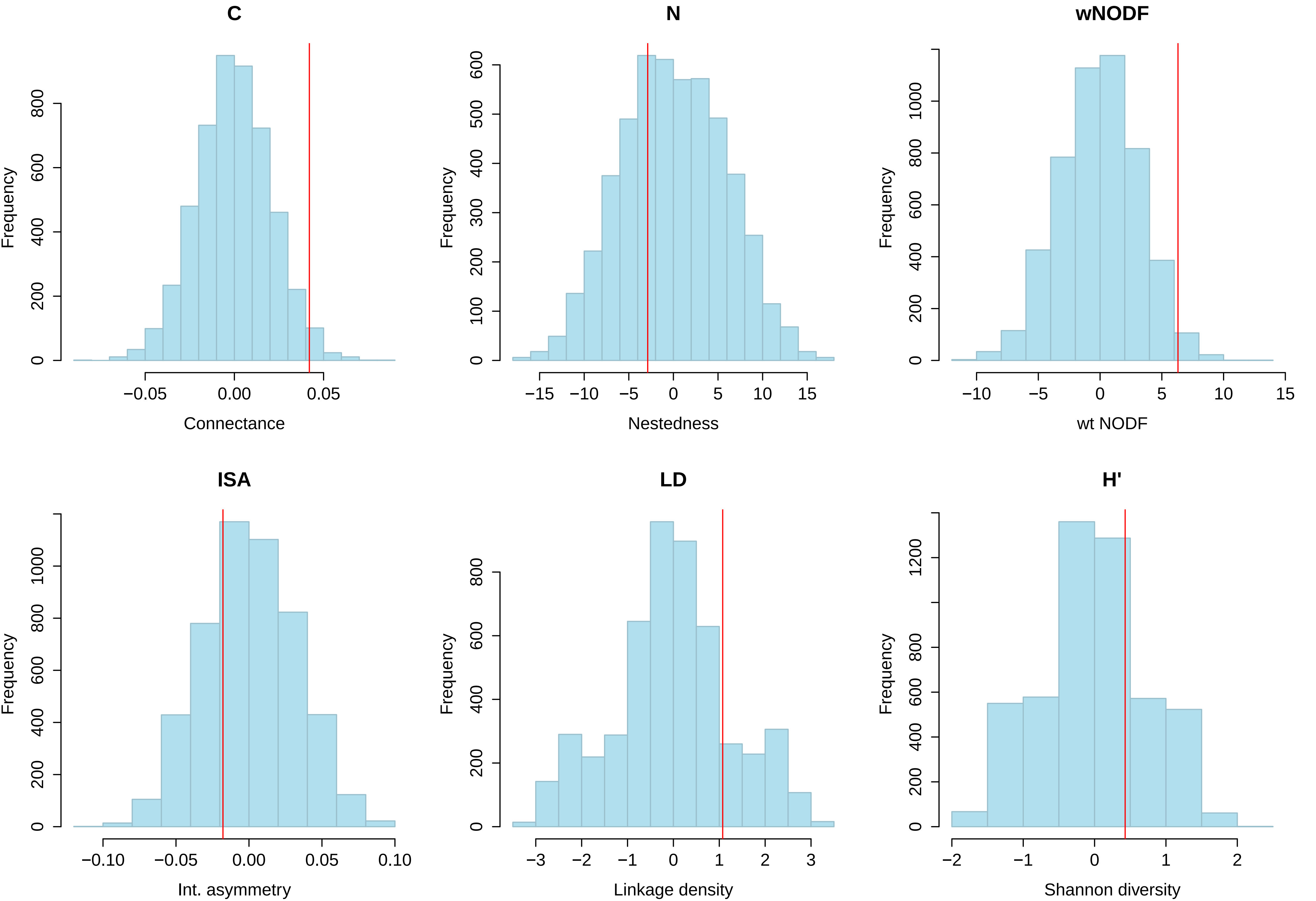
C
Frequency
Connectance
N
Frequency
Nestedness
wNODF
Frequency
wt NODF
ISA
Frequency
Int. asymmetry
LD
Frequency
Linkage density
H'
Frequency
Shannon diversity

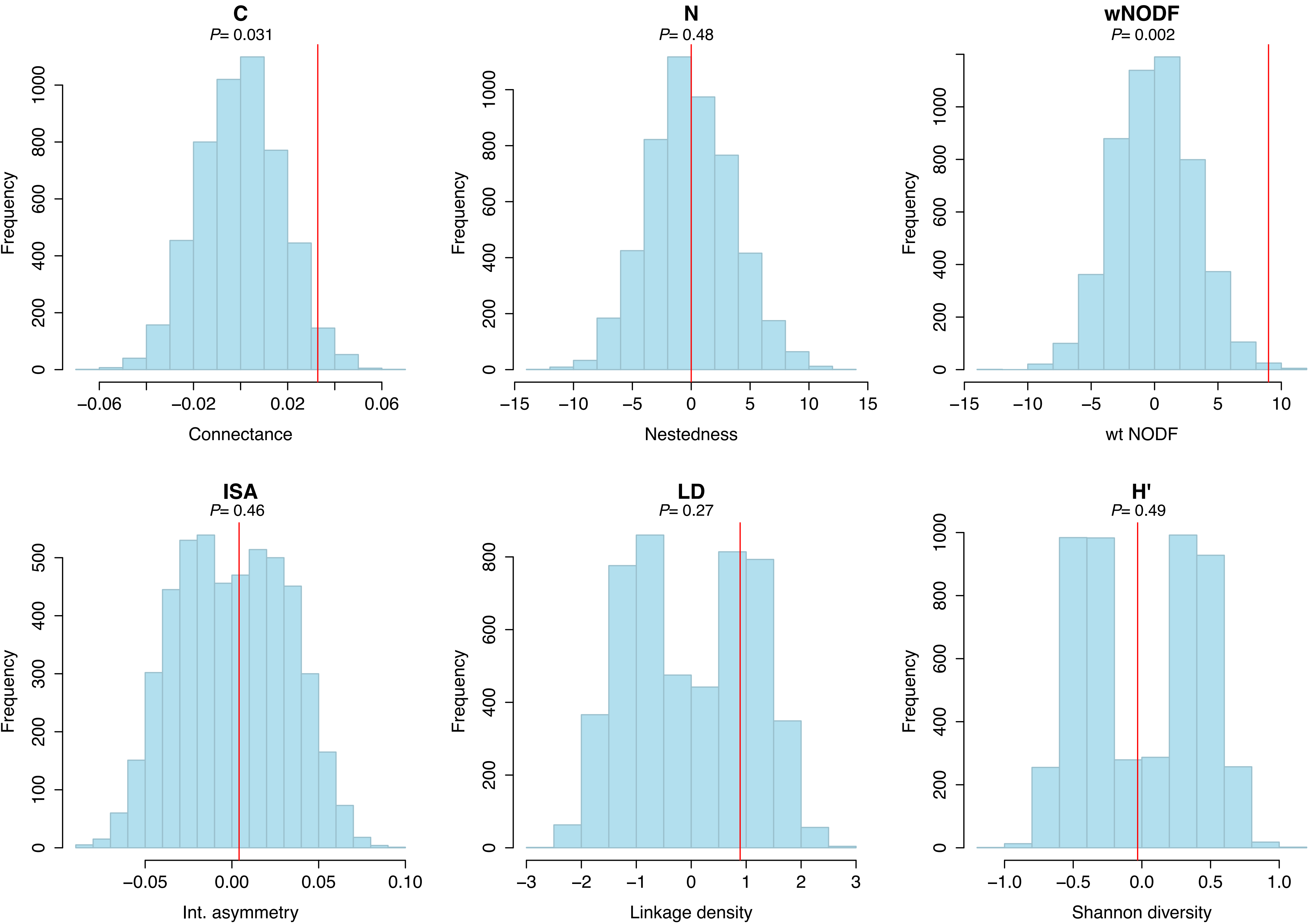
C
P= 0.031
Frequency
Connectance
N
P= 0.48
Nestedness
wNODF
P= 0.002
wt NODF
ISA
P= 0.46
Int. asymmetry
LD
P= 0.27
Linkage density
H'
P= 0.49
Shannon diversity

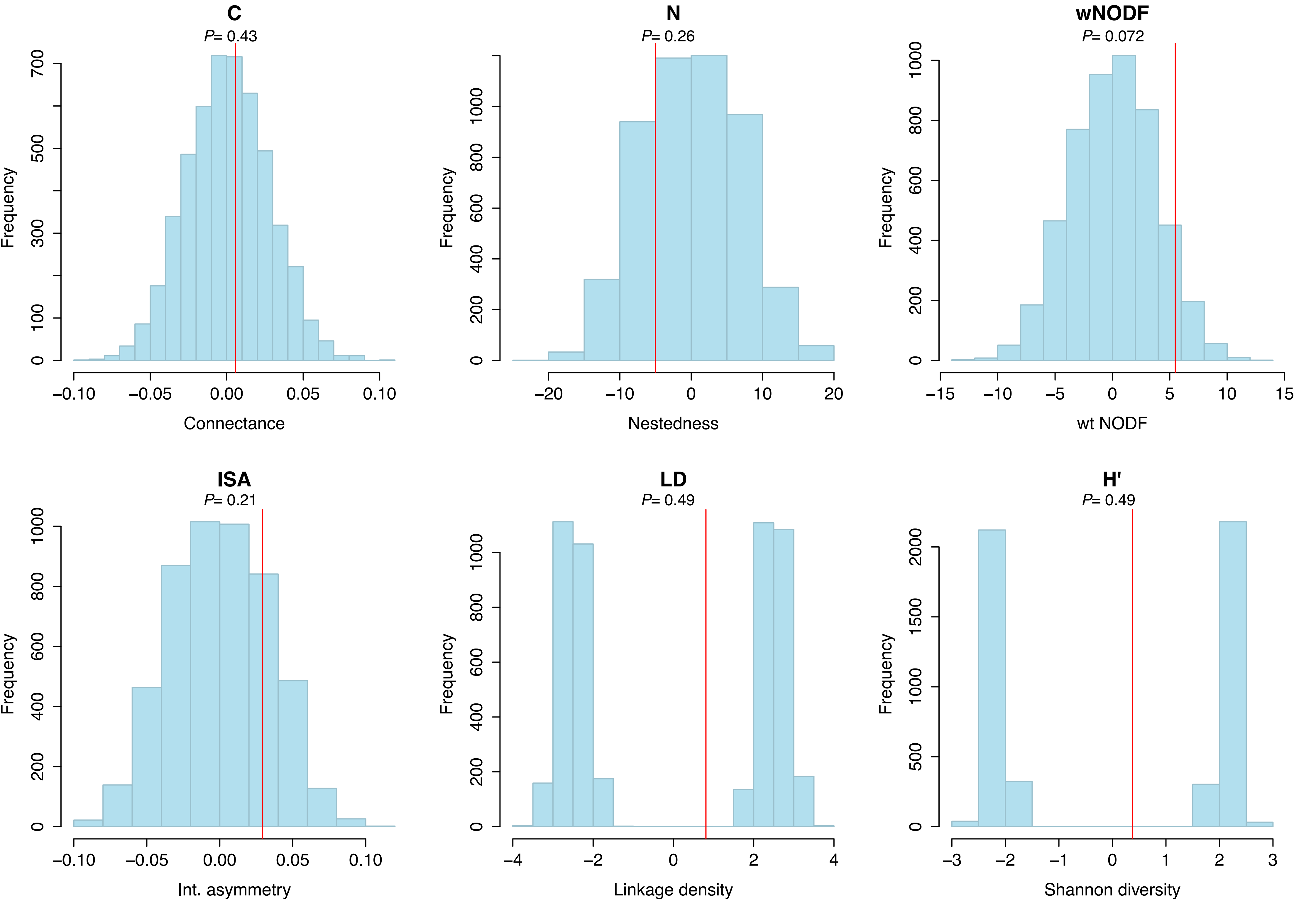
C
P= 0.43
Frequency
Connectance
N
P= 0.26
Frequency
Nestedness
wNODF
P= 0.072
Frequency
wt NODF
ISA
P= 0.21
Frequency
Int. asymmetry
LD
P= 0.49
Frequency
Linkage density
H'
P= 0.49
Frequency
Shannon diversity

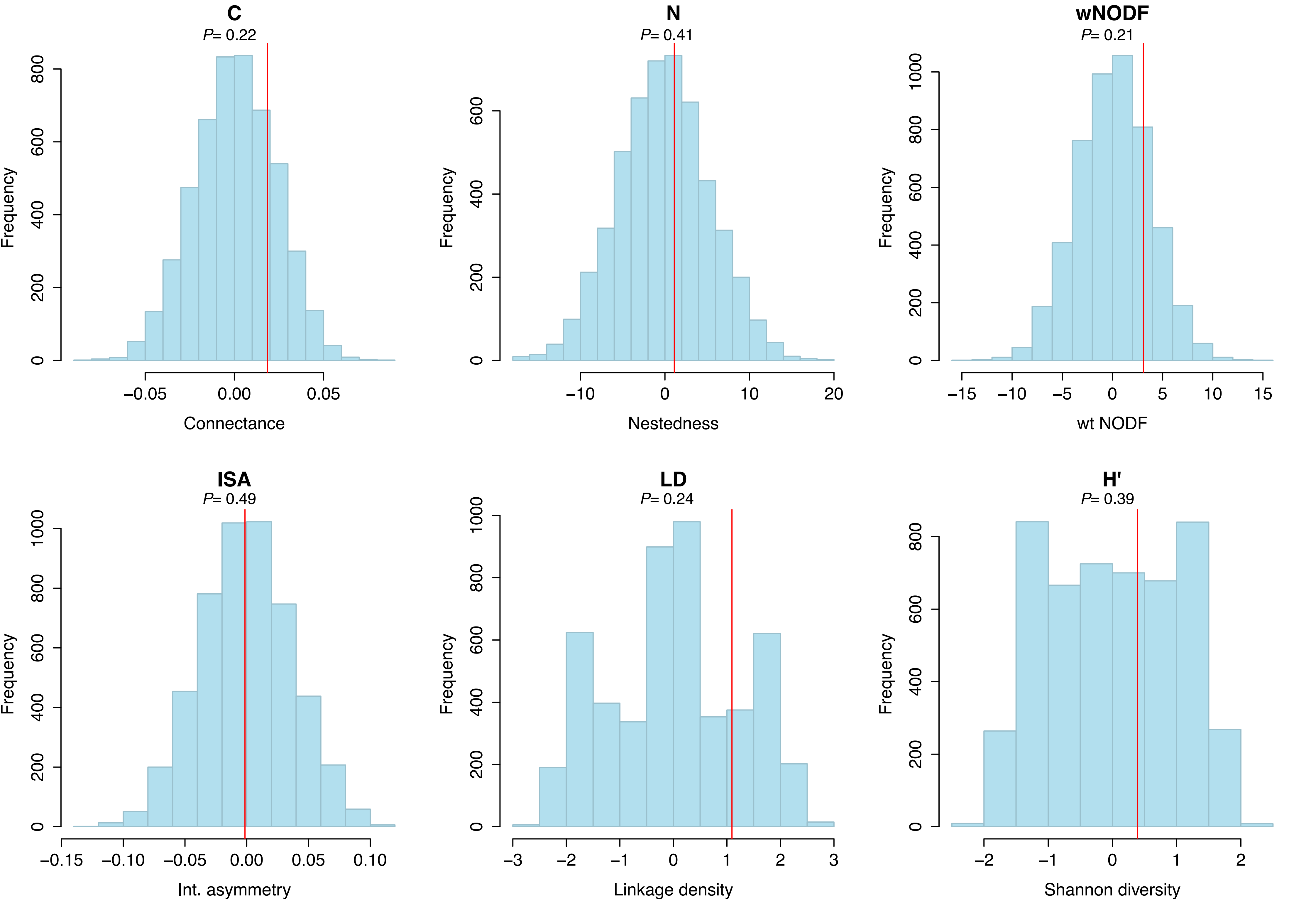
C
P= 0.22
Frequency
Connectance
N
P= 0.41
Nestedness
wNODF
P= 0.21
wt NODF
ISA
P= 0.49
Int. asymmetry
LD
P= 0.24
Linkage density
H'
P= 0.39
Shannon diversity

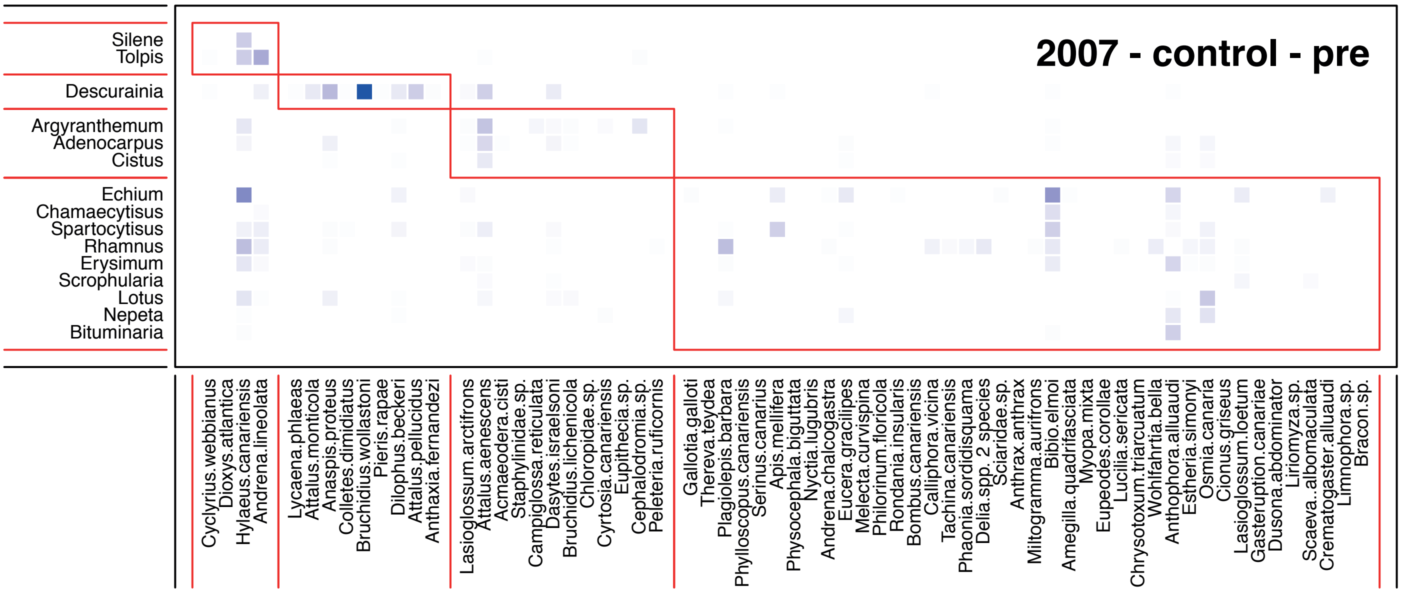
2007 - control - pre
Silene
Tolpis
Descurainia
Argyranthemum
Adenocarpus
Cistus
Echium
Chamaecytisus
Spartocytisus
Rhamnus
Erysimum
Scrophularia
Lotus
Nepeta
Bituminaria
Cyclyrius.webbianus
Dioxys.atlantica
Hylaeus.canariensis
Andrena.lineolata
Lycaena.phlaeas
Attalus.monticola
Anaspis.proteus
Colletes.dimidiatus
Bruchidius.wollastoni
Pieris.rapae
Dilophus.beckeri
Attalus.pellucidus
Anthaxia.fernandezi
Lasioglossum.arctifrons
Attalus.aenescens
Acmaeodera.cisti
Staphylinidae.sp.
Campiglossa.reticulata
Dasytes.israelsoni
Bruchidius.lichenicola
Chloropidae.sp.
Cyrtosia.canariensis
Eupithecia.sp.
Cephalodromia.sp.
Peleteria.ruficornis
Gallotia.galloti
Thereva.teydea
Plagiolepis.barbara
Phylloscopus.canariensis
Serinus.canarius
Apis.mellifera
Physocephala.biguttata
Nyctia.lugubris
Andrena.chalcogastra
Eucera.gracilipes
Melecta.curvispina
Philorinum.floricola
Rondania.insularis
Bombus.canariensis
Calliphora.vicina
Tachina.canariensis
Phaonia.sordidisquama
Delia.spp. 2 species
Sciaridae.sp.
Anthrax.anthrax
Miltogramma.aurifrons
Bibio.elmoi
Amegilla.quadrifasciata
Myopa.mixta
Eupeodes.corollae
Lucilia.sericata
Chrysotoxum.triarcuatum
Wohlfahrtia.bella
Anthophora.alluaudi
Estheria.simonyi
Osmia.canaria
Cionus.griseus
Lasioglossum.loetum
Gasteruption.canariae
Dusona.abdominator
Liriomyza.sp.
Scaeva..albomaculata
Crematogaster.alluaudi
Limnophora.sp.
Bracon.sp.

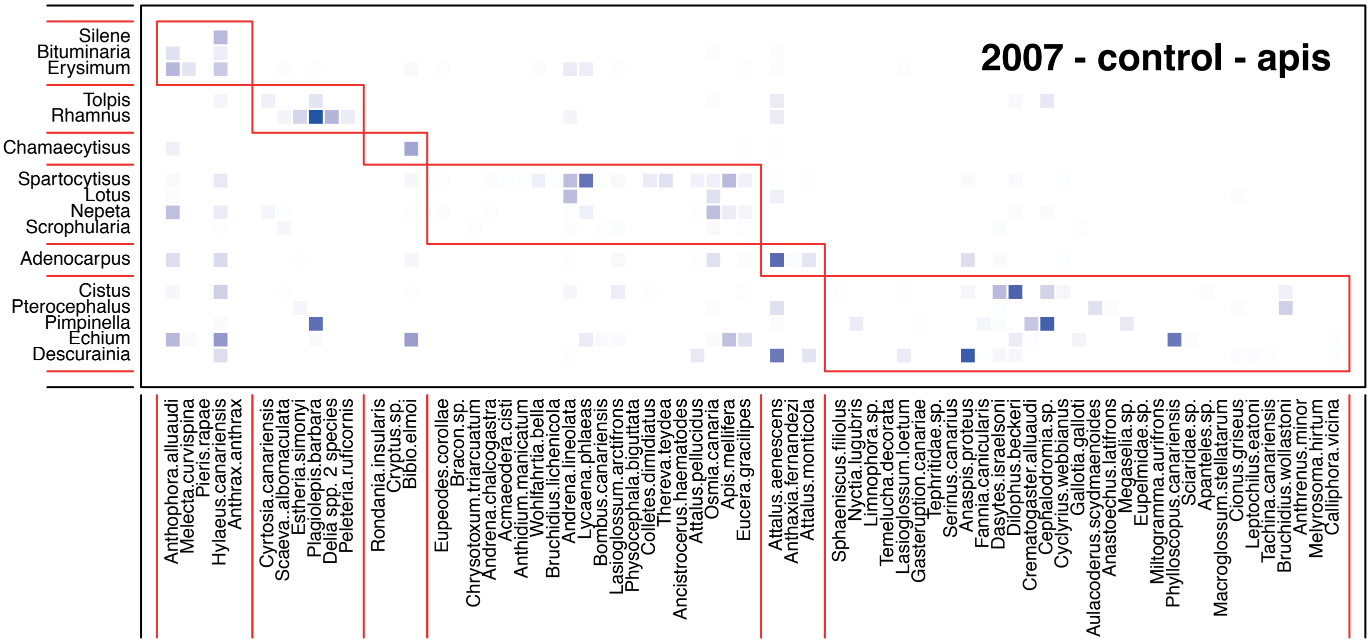
2007 - control - apis
Silene
Bituminaria
Erysimum
Tolpis
Rhamnus
Chamaecytisus
Spartocytisus
Lotus
Nepeta
Scrophularia
Adenocarpus
Cistus
Pterocephalus
Pimpinella
Echium
Descurainia
Anthophora.alluaudi
Melecta.curvispina
Pieris.rapae
Hylaeus.canariensis
Anthrax.anthrax
Cyrtosia.canariensis
Scaeva..albomaculata
Estheria.simonyi
Plagiolepis.barbara
Delia spp. 2 species
Peleteria.ruficornis
Rondania.insularis
Cryptus.sp.
Bibio.elmoi
Eupeodes.corollae
Bracon.sp.
Chrysotoxum.triarcuatum
Andrena.chalcogastra
Acmaeodera.cisti
Anthidium.manicatum
Wohlfahrtia.bella
Bruchidius.lichenicola
Andrena.lineolata
Lycaena.phlaeas
Bombus.canariensis
Lasioglossum.arctifrons
Physocephala.biguttata
Colletes.dimidiatus
Thereva.teydea
Ancistrocerus.haematodes
Attalus.pellucidus
Osmia.canaria
Apis.mellifera
Eucera.gracilipes
Attalus.aenescens
Anthaxia.fernandezi
Attalus.monticola
Sphaeniscus.filiolus
Nyctia.lugubris
Limnophora.sp.
Temelucha.decorata
Lasioglossum.loetum
Gasteruption.canariae
Tephritidae.sp.
Serinus.canarius
Anaspis.proteus
Fannia.canicularis
Dasytes.israelsoni
Dilophus.beckeri
Crematogaster.alluaudi
Cephalodromia.sp.
Cyclyrius.webbianus
Gallotia.galloti
Aulacoderus.scydmaenoides
Anastoechus.latifrons
Megaselia.sp.
Eupelmidae.sp.
Miltogramma.aurifrons
Phylloscopus.canariensis
Sciaridae.sp.
Apanteles.sp.
Macroglossum.stellatarum
Cionus.griseus
Leptochilus.eatoni
Tachina.canariensis
Bruchidius.wollastoni
Anthrenus.minor
Melyrosoma.hirtum
Calliphora.vicina

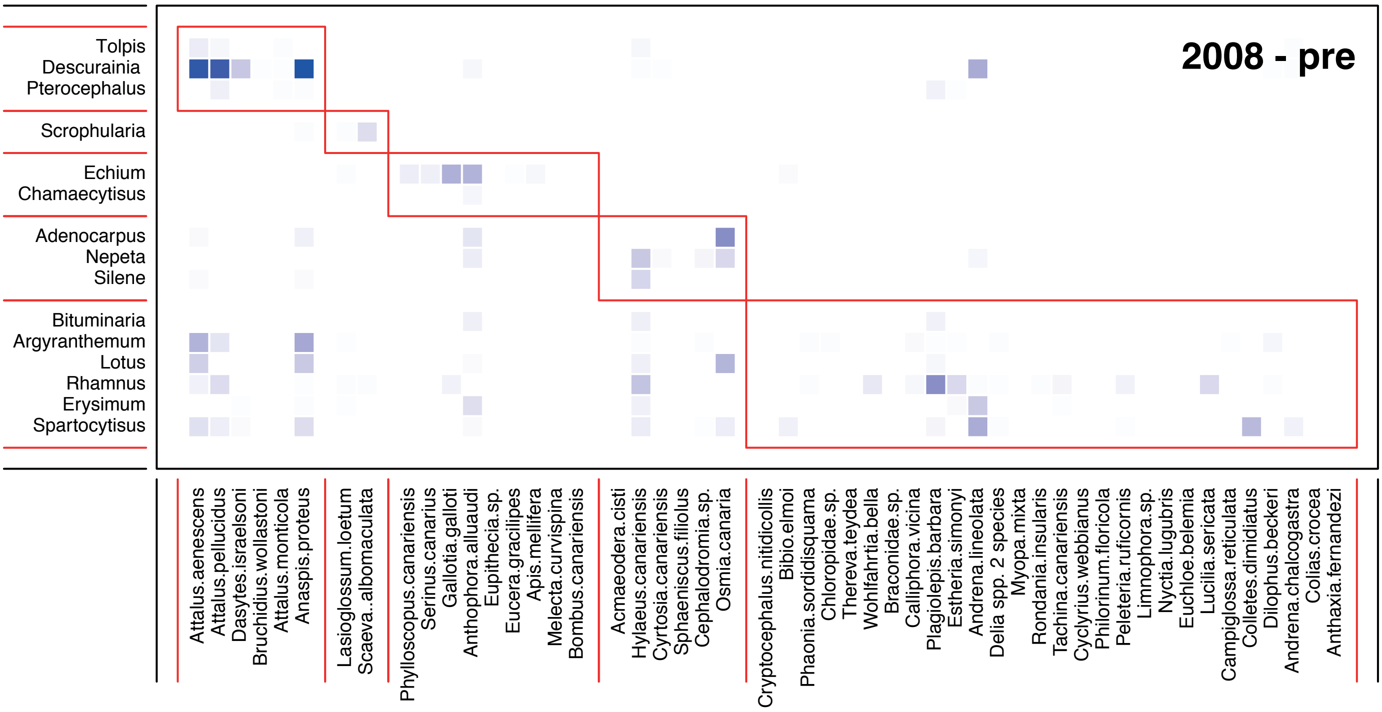
2008 - pre
Tolpis
Descurainia
Pterocephalus
Scrophularia
Echium
Chamaecytisus
Adenocarpus
Nepeta
Silene
Bituminaria
Argyranthemum
Lotus
Rhamnus
Erysimum
Spartocytisus
Attalus.aenescens
Attalus.pellucidus
Dasytes.israelsoni
Bruchidius.wollastoni
Attalus.monticola
Anaspis.proteus
Lasioglossum.loetum
Scaeva..albomaculata
Phylloscopus.canariensis
Serinus.canarius
Gallotia.galloti
Anthophora.alluaudi
Eupithecia.sp.
Eucera.gracilipes
Apis.mellifera
Melecta.curvispina
Bombus.canariensis
Acmaeodera.cisti
Hylaeus.canariensis
Cyrtosia.canariensis
Sphaeniscus.filiolus
Cephalodromia.sp.
Osmia.canaria
Cryptocephalus.nitidicollis
Bibio.elmoi
Phaonia.sordidisquama
Chloropidae.sp.
Thereva.teydea
Wohlfahrtia.bella
Braconidae.sp.
Calliphora.vicina
Plagiolepis.barbara
Estheria.simonyi
Andrena.lineolata
Delia spp. 2 species
Myopa.mixta
Rondania.insularis
Tachina.canariensis
Cyclyrius.webbianus
Philorinum.floricola
Peleteria.ruficornis
Limnophora.sp.
Nyctia.lugubris
Euchloe.belemia
Lucilia.sericata
Campiglossa.reticulata
Colletes.dimidiatus
Dilophus.beckeri
Andrena.chalcogastra
Colias.crocea
Anthaxia.fernandezi

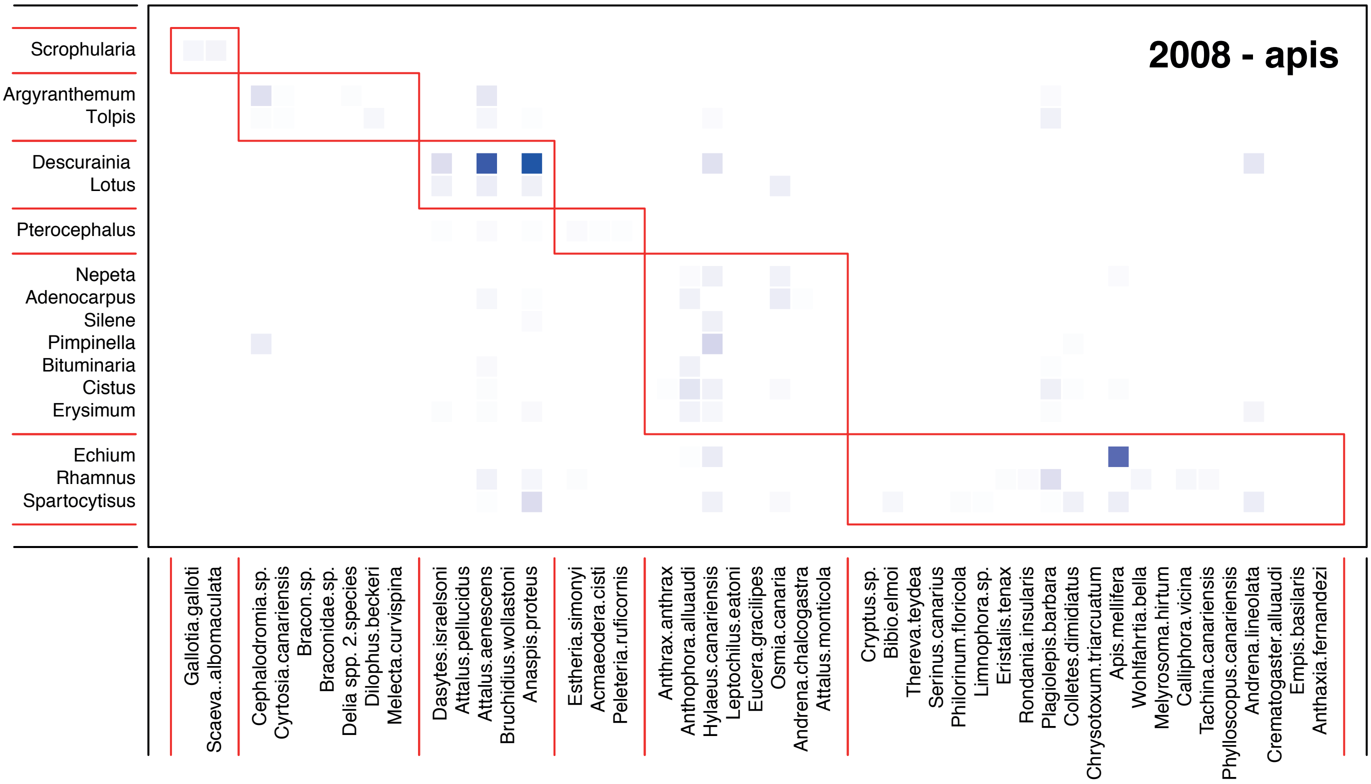
2008 - apis
Scrophularia
Argyranthemum
Tolpis
Descurainia
Lotus
Pterocephalus
Nepeta
Adenocarpus
Silene
Pimpinella
Bituminaria
Cistus
Erysimum
Echium
Rhamnus
Spartocytisus
Gallotia.galloti
Scaeva..albomaculata
Cephalodromia.sp.
Cyrtosia.canariensis
Bracon.sp.
Braconidae.sp.
Delia spp. 2.species
Dilophus.beckeri
Melecta.curvispina
Dasytes.israelsoni
Attalus.pellucidus
Attalus.aenescens
Bruchidius.wollastoni
Anaspis.proteus
Estheria.simonyi
Acmaeodera.cisti
Peleteria.ruficornis
Anthrax.anthrax
Anthophora.alluaudi
Hylaeus.canariensis
Leptochilus.eatoni
Eucera.gracilipes
Osmia.canaria
Andrena.chalcogastra
Attalus.monticola
Cryptus.sp.
Bibio.elmoi
Thereva.teydea
Serinus.canarius
Philorinum.floricola
Limnophora.sp.
Eristalis.tenax
Rondania.insularis
Plagiolepis.barbara
Colletes.dimidiatus
Chrysotoxum.triarcuatum
Apis.mellifera
Wohlfahrtia.bella
Melyrosoma.hirtum
Calliphora.vicina
Tachina.canariensis
Phylloscopus.canariensis
Andrena.lineolata
Crematogaster.alluaudi
Empis.basilaris
Anthaxia.fernandezi

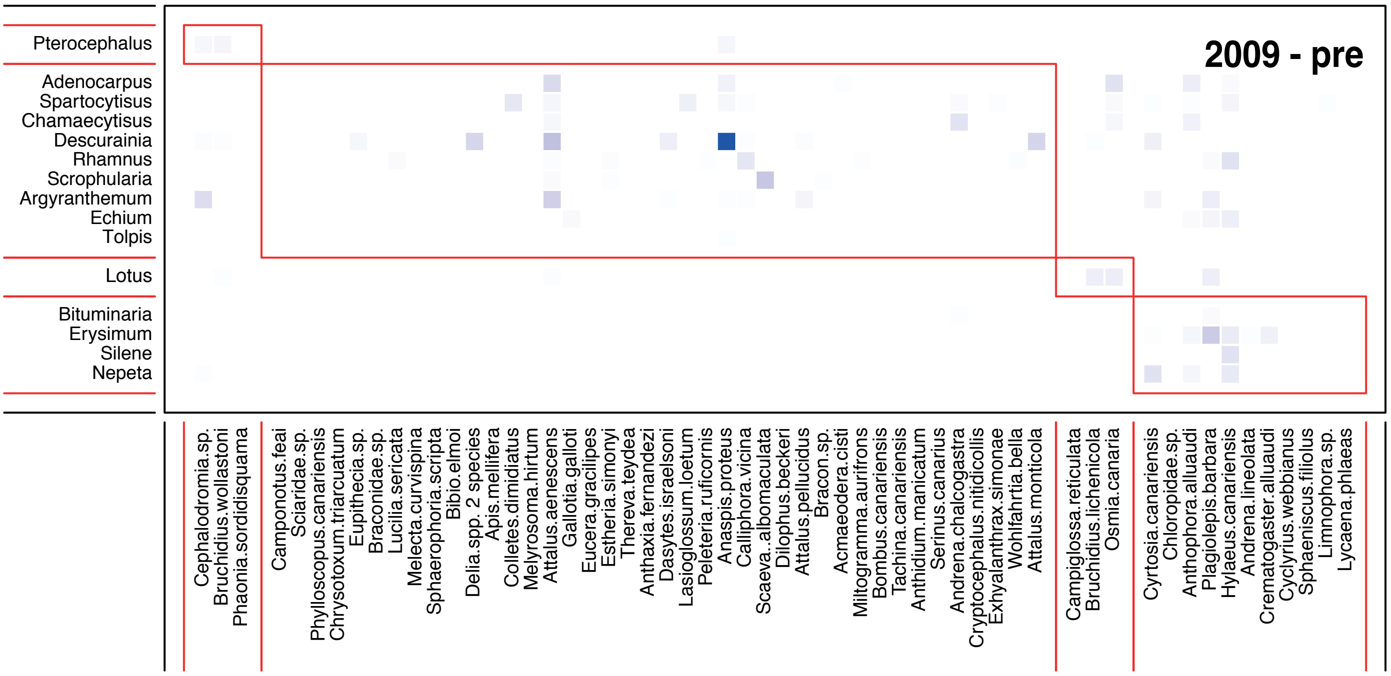
2009 - pre

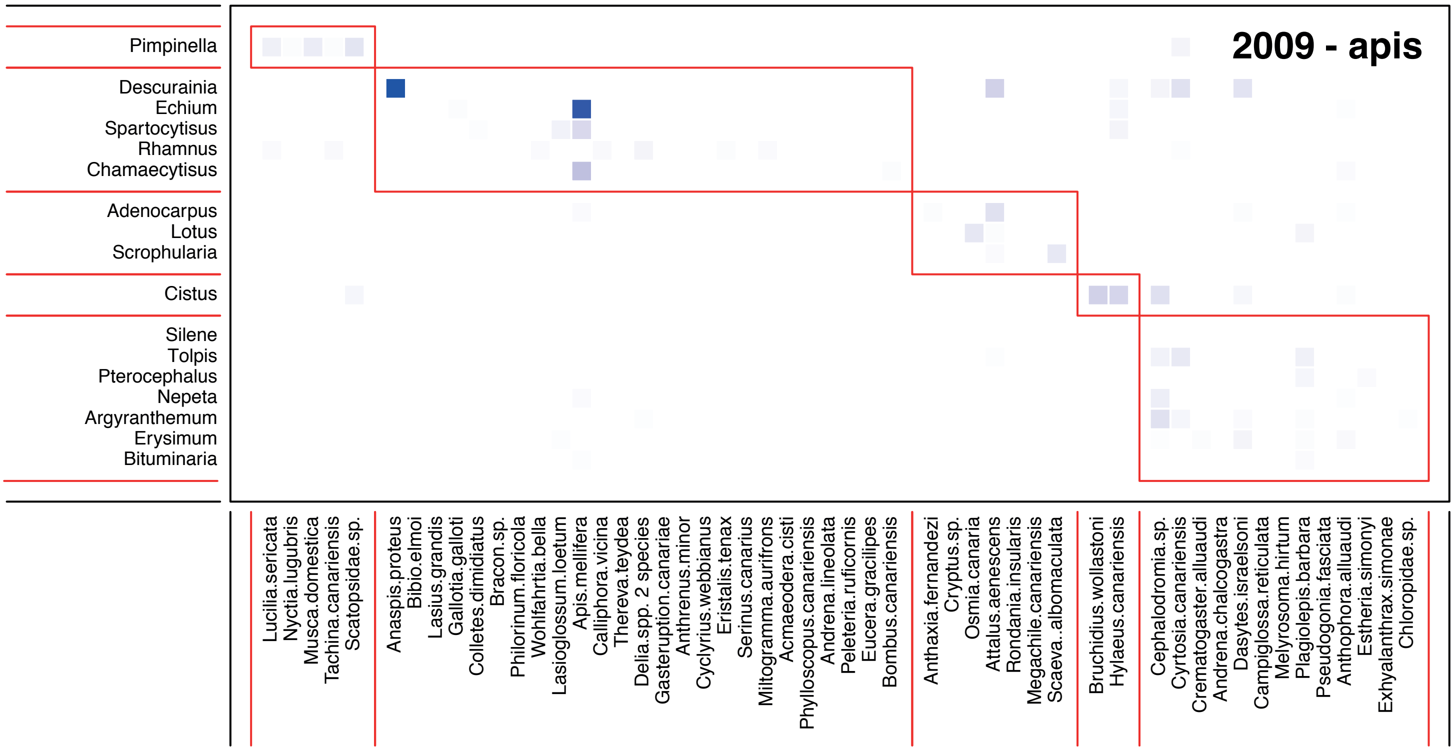
2009 - apis

2008-2009 - pre

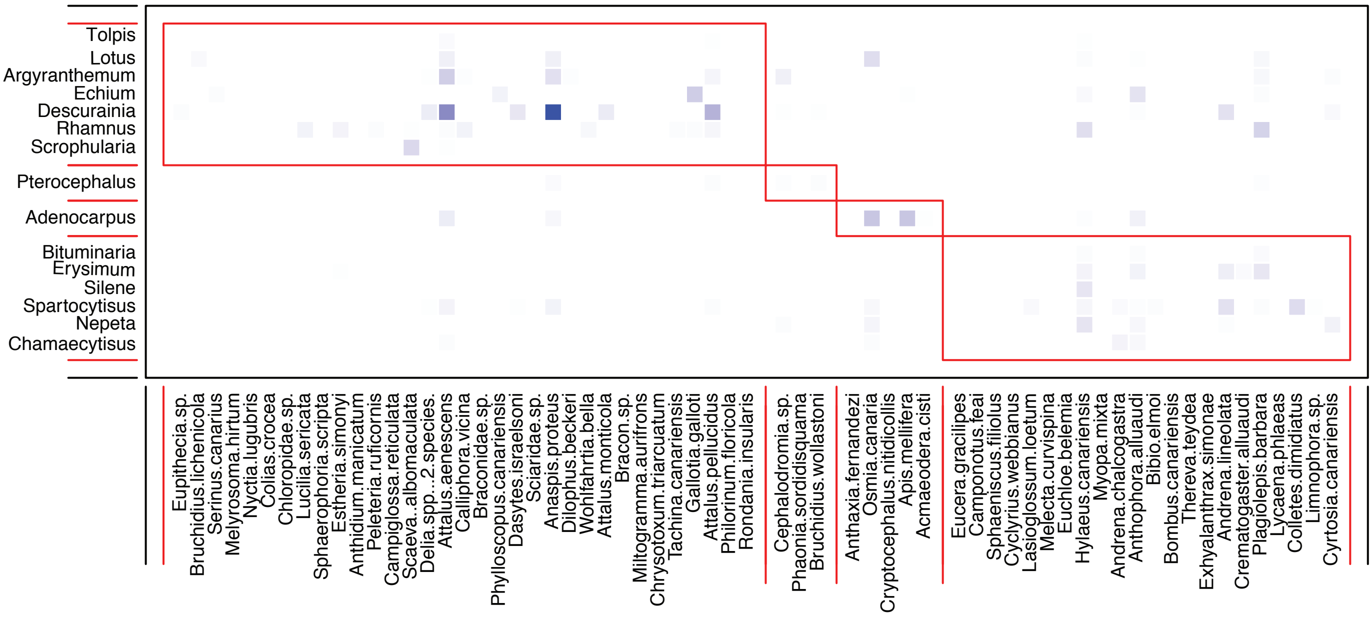

2008-2009 - apis

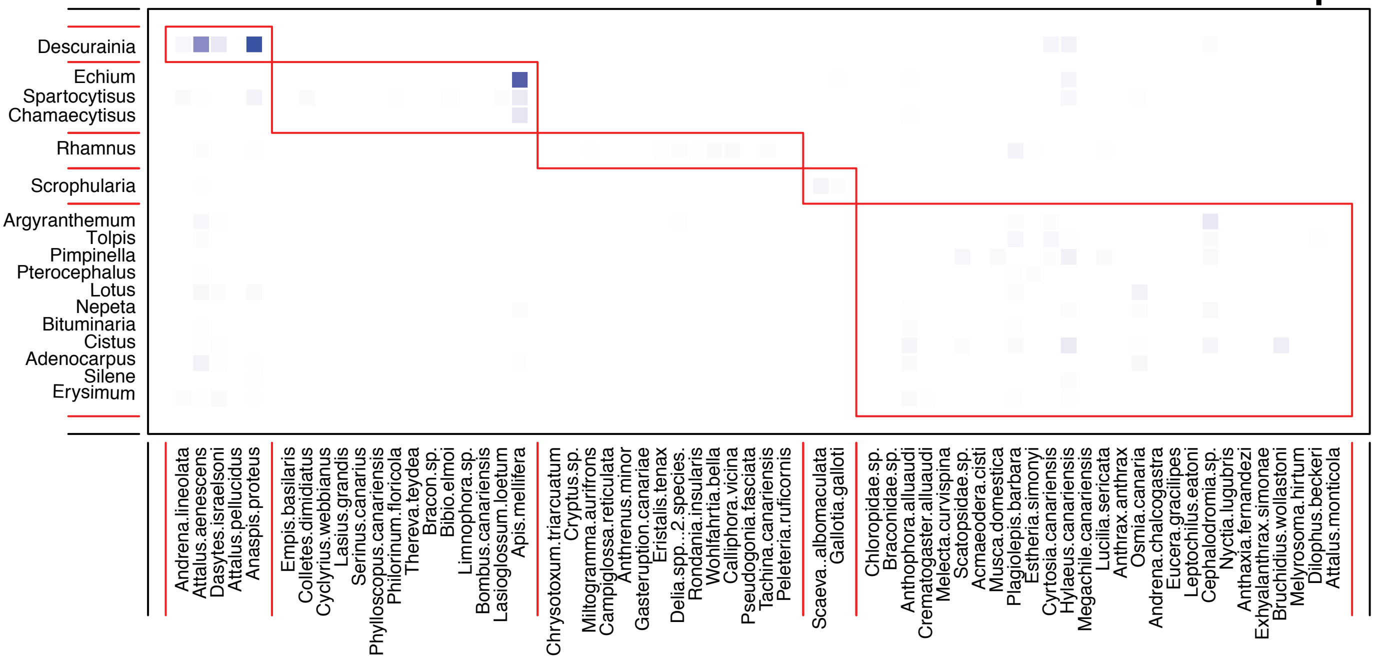

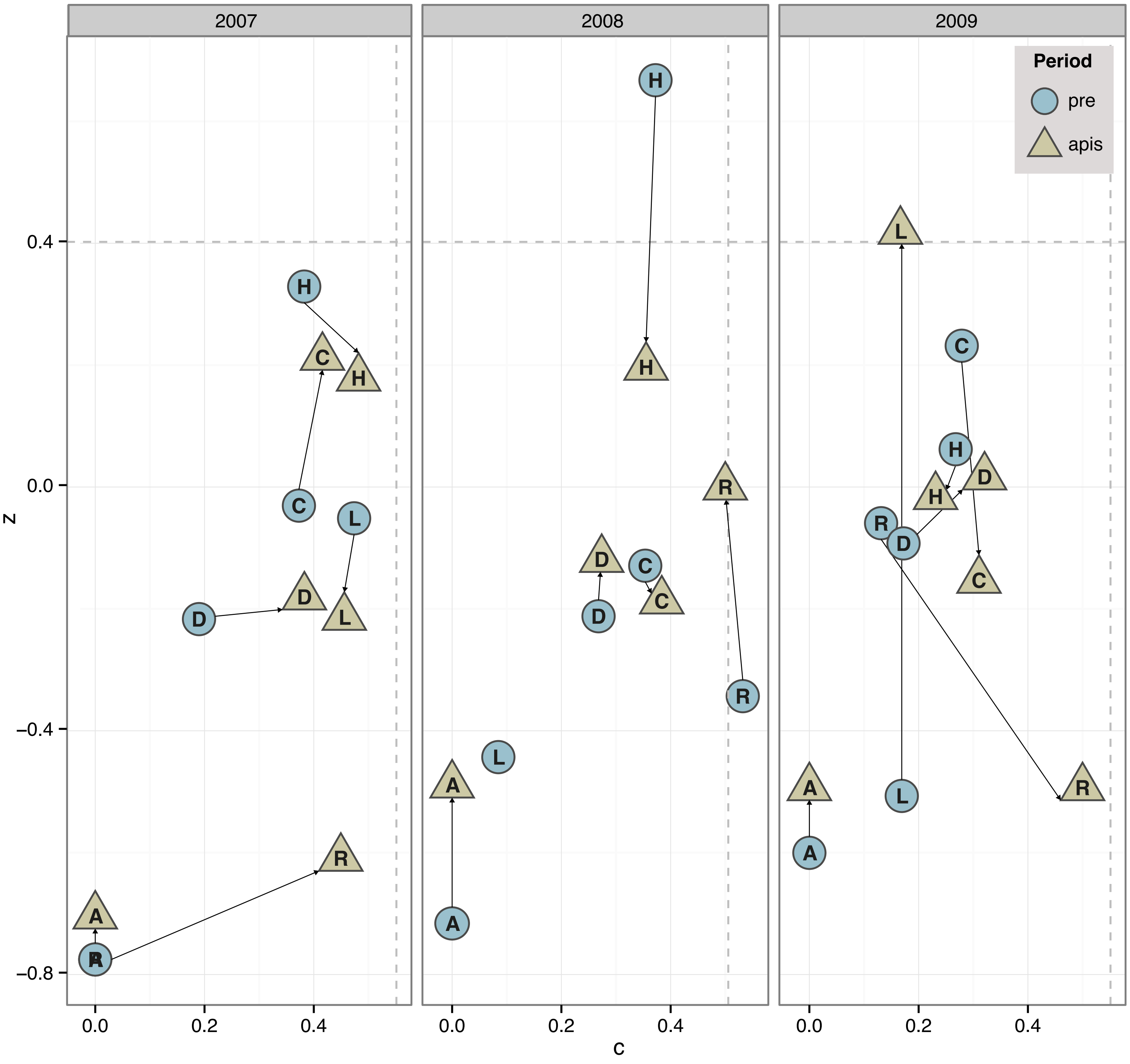
2007
2008
2009
Period
pre
apis
z
c
0.4
0.0
−0.4
−0.8
0.0
0.2
0.4
H
C
D
L
R
A

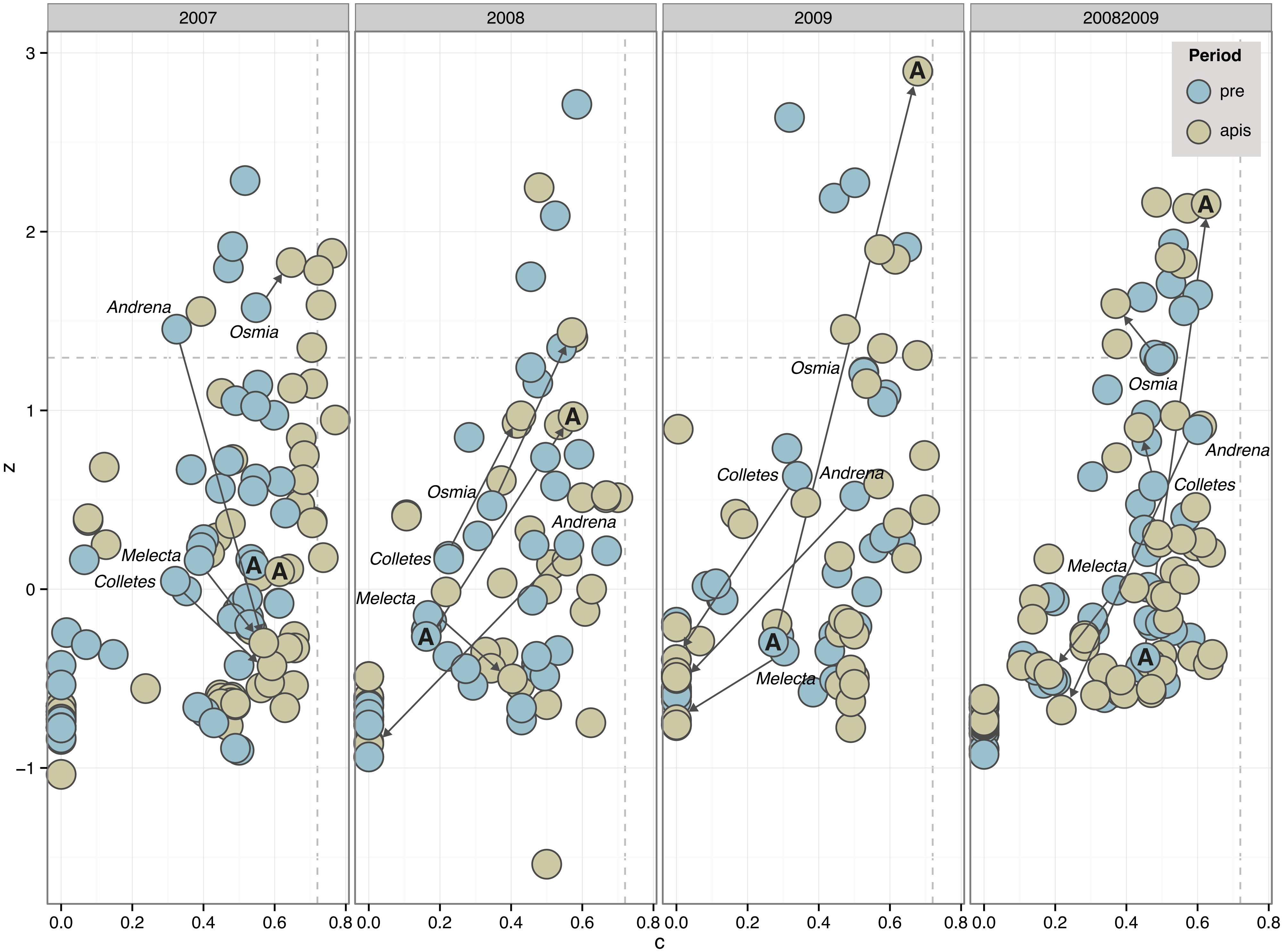

2007
2008
2009
20082009
Period
pre
apis
Andrena
Osmia
Melecta
Colletes
A
z
c
3
2
1
0
−1
0.0
0.2
0.4
0.6
0.8

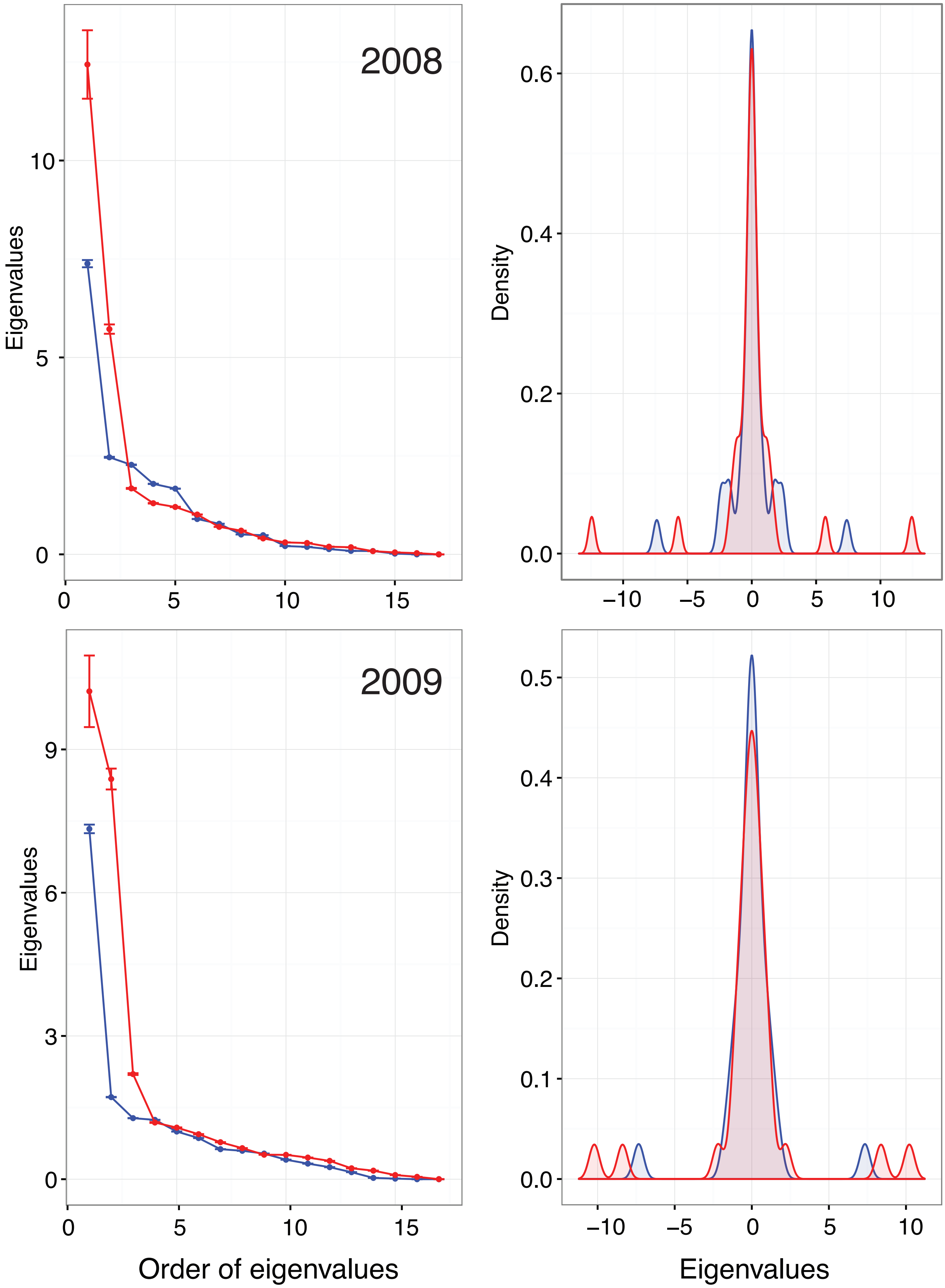

2008
2009
Eigenvalues
Density
Order of eigenvalues
Eigenvalues

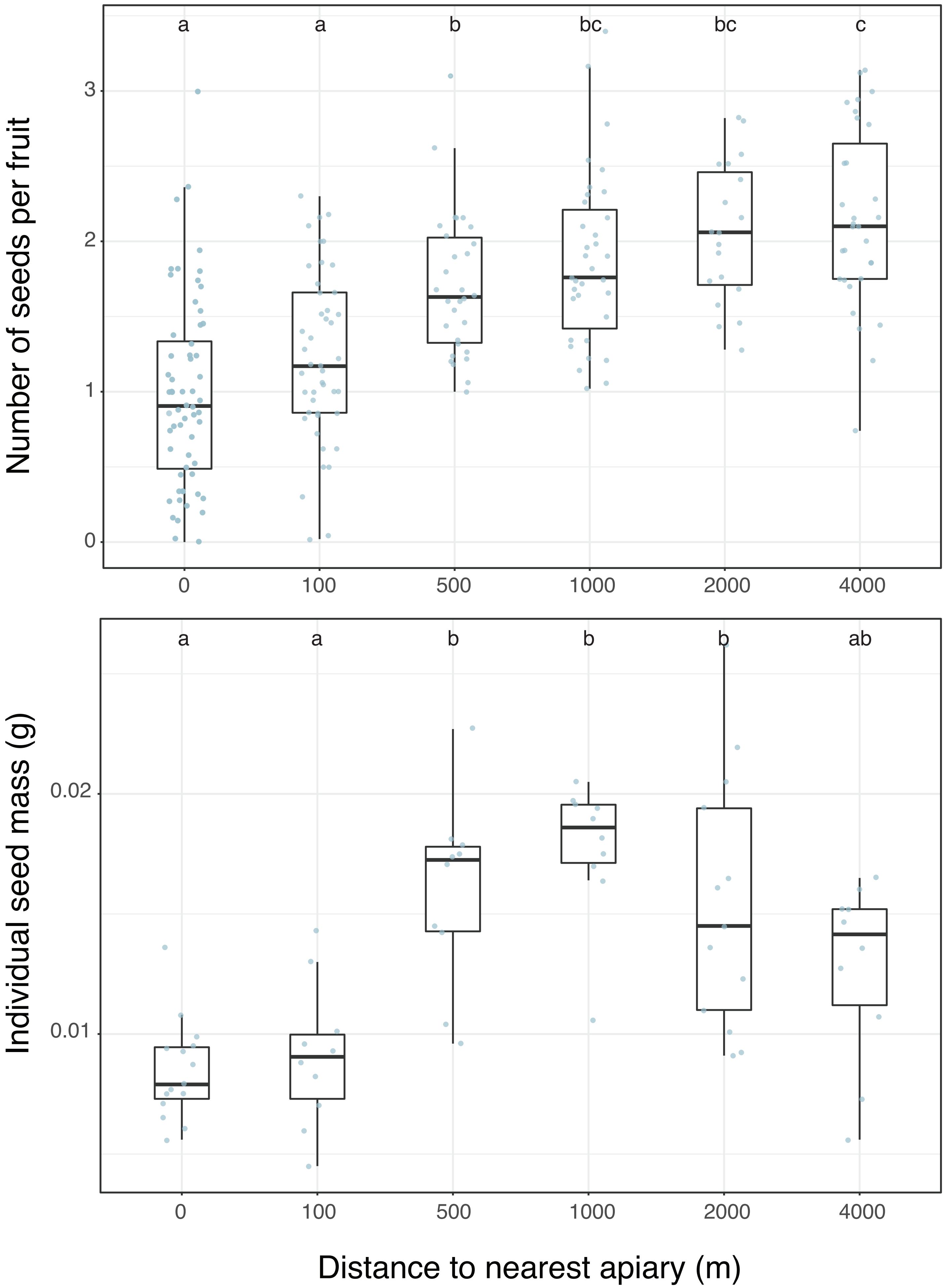

a
a
b
bc
bc
c
Number of seeds per fruit
0
1
2
3
0
100
500
1000
2000
4000
a
a
b
b
b
ab
Individual seed mass (g)
0.01
0.02
0
100
500
1000
2000
4000
Distance to nearest apiary (m)

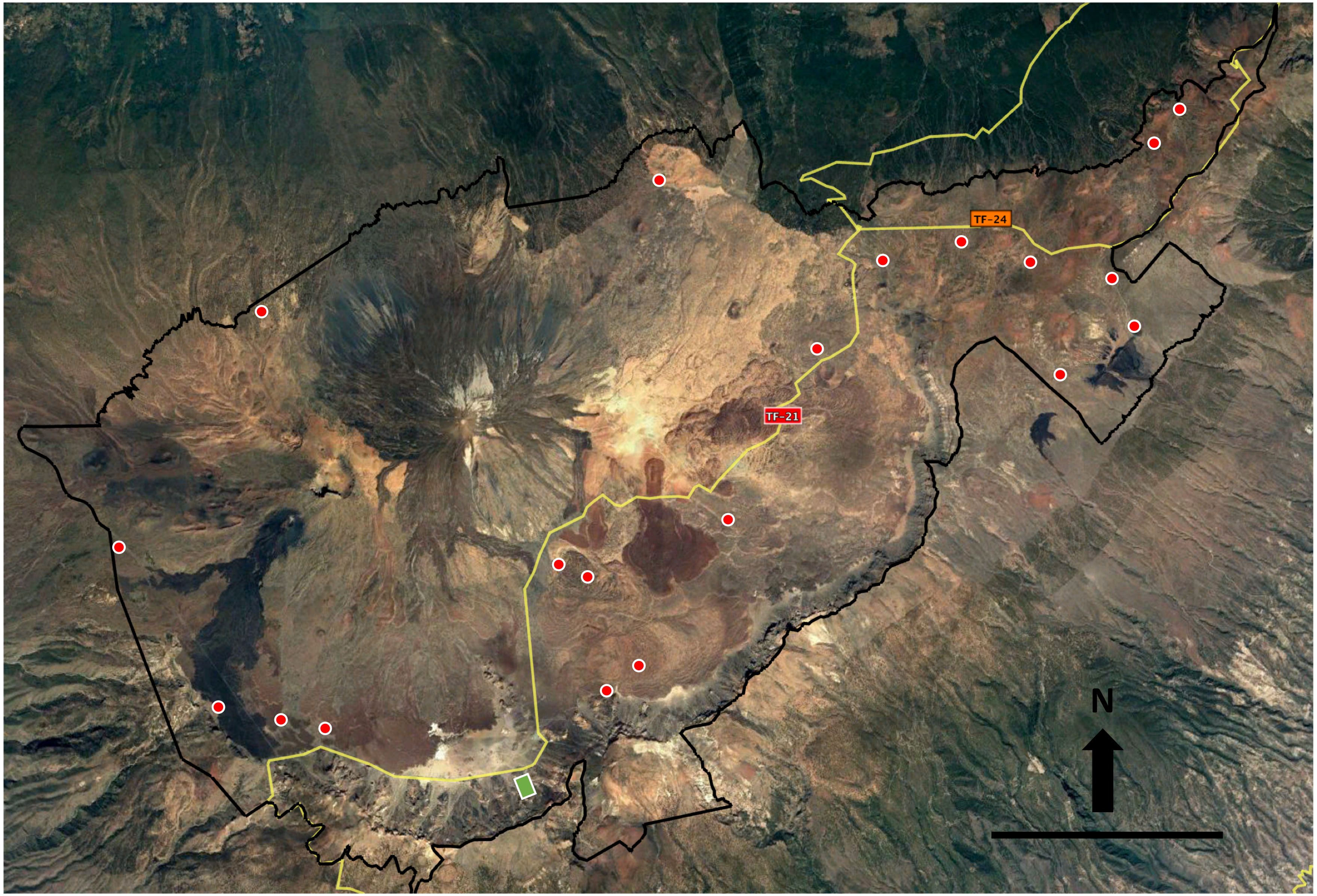
TF-24
TF-21
N
